# Molecular Targeting of the BRAF Proto-Oncogene/Mitogen-Activated Protein Kinase (MAPK) Pathway across Cancers

**DOI:** 10.3390/ijms25010624

**Published:** 2024-01-03

**Authors:** Khine S. Shan, Tauseef U. Rehman, Stan Ivanov, Gelenis Domingo, Luis E. Raez

**Affiliations:** 1 Memorial Health Care, Division of Hematology and Oncology, Pembroke Pines, FL 33328, USA; trehman@mhs.net (T.U.R.); sianov@mhs.net (S.I.); gdomingo@mhs.net (G.D.); 2 Memorial Health Care, Thoracic Oncology Program, Pembroke Pines, FL 33328, USA; lraez@mhs.net

**Keywords:** BRAF, MEK, ERK, MAPK, molecular profiling, targeted therapy, tumor agnostic, personalized medicine

## Abstract

The mitogen-activated protein kinase (MAPK) pathway is essential for cellular proliferation, growth, and survival. Constitutive activation of this pathway by BRAF mutations can cause downstream activation of kinases, leading to uncontrolled cellular growth and carcinogenesis. Therefore, inhibition of BRAF and the downstream substrate MEK has been shown to be effective in controlling tumor growth and proliferation. Over the last decade, several BRAF and MEK inhibitors have been investigated, ranging from primarily melanoma to various cancer types with BRAF alterations. This subsequently led to several Food and Drug Administration (FDA) approvals for BRAF/MEK inhibitors for melanoma, non-small cell lung cancer, anaplastic thyroid cancer, colorectal cancer, histiocytosis neoplasms, and finally, tumor-agnostic indications. Here, this comprehensive review will cover the developments of BRAF and MEK inhibitors from melanomas to tumor-agnostic indications, novel drugs, challenges, future directions, and the importance of those drugs in personalized medicine.

## 1. Introduction

The mitogen-activated protein kinase (MAPK) pathway is important for several vital cellular functions, such as differentiation, proliferation, survival, autophagy, and apoptosis [1]. Activation of any step in the MAPK pathway (RAS/RAF/MEK/ERK) can lead to downstream activation and carcinogenesis. BRAF is one of the three isoforms of the RAF protein kinase. BRAF and MEK inhibitors have been developed for the treatment of various types of cancers due to their inhibition of the MAPK pathway and subsequent inhibition of cancer growth. Targeting the BRAF/MEK pathway has become one of the recent advancements in precision medicine. This review will mainly discuss BRAF inhibitors (vemurafenib, dabrafenib, and encorafenib) and MEK 1/2 inhibitors (trametinib, cobimetinib, and binimetinib), their indications across various types of cancers, combinations with immunotherapy, associated toxicities, resistance mechanisms, as well as novel drugs and future perspectives.

## 2. MAPK Pathway

**Figure 1:**
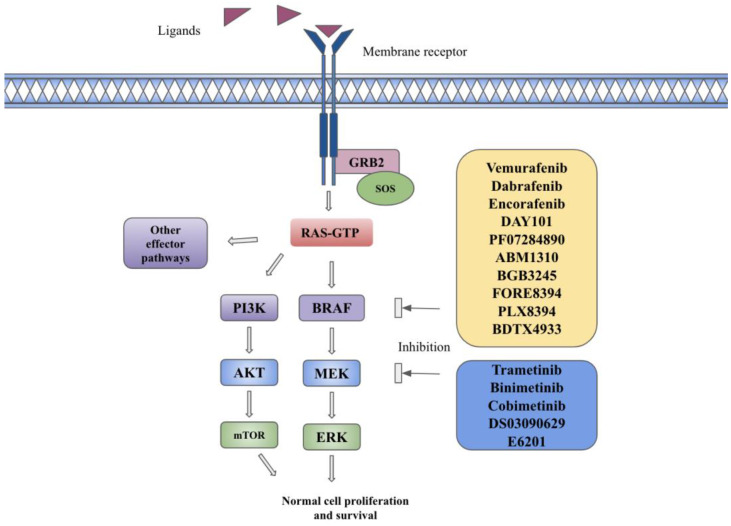
The BRAF signaling pathway and targeted therapies in the BRAF pathway. Description: Binding of ligands to transmembrane receptors, such as FGFR or EGFR, leads to receptor dimerization and phosphorylation of kinases, which leads to the recruitment of growth factor receptor-bound protein 2 (GRB2) to the phosphorylated receptor. The attachment of Son of Sevenless (SOS—GTP exchange factor) to GRB2 enables the activation of RAS-GDP to RAS-GTP. The active form of RAS-GTP causes downstream activation of MAPK, PI3K, and other effector pathways. BRAF inhibitors in yellow box and MEK inhibitors in blue box inhibit BRAF and MEK substrates and inhibit downstream pathway activation.

The MAPK pathway is composed of cytoplasmic serine/threonine and tyrosine kinases, including RAS, RAF, MEK, and ERK [1]. RAS is a GTPase that includes three isoforms encoded by *HRAS*, *NRAS*, or *KRAS* genes [1]. RAF is a protein kinase with three isoforms encoded by the *ARAF*, *BRAF*, and *CRAF* genes [1]. Each RAF isoform is composed of three conserved regions, including the RAS binding domain (CR1), regulatory domain (CR2), and catalytic kinase domain (CR3), as well as isoform-specific domains responsible for embryological functions [2]. BRAF activation is solely dependent on RAS, but BRAF itself is a co-activating factor for CRAF and is a more potent activator of MEK due to its higher affinity for this substrate [2]. MEK proteins consist of three regions, including an N-terminal region for binding its substrate, a core kinase domain, and a C-terminal region [2]. Binding of membrane tyrosine kinase receptors such as fibroblast growth factor receptor (FGFR) or epidermal growth factor receptor (EGFR) to their specific ligands (FGF or EGF) leads to their dimerization and autophosphorylation, resulting in intracellular signaling. It leads to the recruitment of growth factor receptor-bound protein 2 (GRB2) to the phosphorylated receptor. The attachment of Son of Sevenless (SOS—GTP exchange factor) to GRB2 enables the activation of RAS-GDP to RAS-GTP. The active form of RAS-GTP indirectly leads to fixation, dimerization, and phosphorylation of RAF via the SRC kinase family (SKF) and Casein Kinase 2 (CK2) at the plasma membrane. RAF proteins further phosphorylate and activate MEK 1/2 on serines 218 and 222, which in turn lead to phosphorylation and activation of ERK1 on threonine 202 and tyrosine 204 and ERK2 on threonine 185 and tyrosine 187 [1,3]. The activated ERK proteins cause phosphorylation of a variety of substrates as well as enter the nucleus via importin 7 and phosphorylate multiple transcription factors involved in cellular growth, proliferation, and evasion from apoptosis [3]. The MAPK pathway is described in Figure 1.

## 3. Alterations in MAPK Pathway Causing Carcinogenesis

The most common alterations in the MAPK pathway are *RAS*, *BRAF*, and, less frequently, *MEK* and *CRAF* alterations. *BRAF* mutations can be divided into three classes (I, II, and III) depending on their RAS dependency and the activity of the catalytic domain [2]. The *BRAF* proto-oncogene is located on chromosome 7 (7q34), and *BRAF* gene-activating mutations are present in about 7% of human cancers [4]. Class I *BRAF* mutations, which include *BRAFV600 E/K/D/M/R*, with 90% being *BRAFV600E*, have high kinase activity even in their monomer state without RAS signaling (RAS-independent) [2,4,5]. Class II *BRAF* mutations include non-*V600* mutations, including *K601E*, *K601N*, *K601T*, and *L597Q* mutations at the *BRAF* activation segment; *G464*, *G469A*, *G469V*, and *G469R* mutations within the P-loop; and chromosomal alterations such as fusions and deletions [4]. Class II mutations are also RAS-independent, but they have intermediate kinase activity as monomers and require dimer formation [2,5]. Class III *BRAF* mutations, including *D954N*, *N581S*, *G466V*, *D594G*, *G466E*, and *G596D* point mutations, are dependent on RAS signaling and need dimerization with other wild-type CRAF isoforms to fully function [2,4,5]. Class I and II mutations do not require upstream RAS pathway activation and are mutually exclusive with other co-concurrent mutations, while Class III mutations require upstream activation and often co-exist with upstream RAS mutations [2].

Class I *BRAF* alterations are the most prevalent, with 53% present in *BRAF*-altered samples in the AACR project GENIE (Genomics Evidence Neoplasia Information Exchange) analysis, and current approved BRAF inhibitors such as vemurafenib, dabrafenib, and encorafenib mainly inhibit class I mutations [5,6]. The most common activating *BRAF* mutation is the *BRAFV600E* hotspot mutation, which is caused by the transversion of a thymine (T) to an adenine (A) at position 1799 in exon 15, leading to the replacement of the amino acid valine with glutamic acid and constitutive activation of the BRAF kinase domain without being activated by RAS [1]. Class I BRAF inhibitors competitively bind to the ATP-binding pocket of RAF kinase, which stabilizes kinase in its active conformation, thus forcing the protein to take an inactive one and leading to downstream MAPK pathway inhibition, cell cycle arrest, and apoptosis [4,7,8].

MEK mutations are also divided into three classes depending on RAF dependency, including RAF-independent, RAF-regulated, and RAF-dependent alterations. RAF-independent MEK alterations are usually in-frame deletions resulting in hyperactive MEK domains, whereas RAF-regulated and -dependent MEK mutations require RAF phosphorylation for full function [2]. Current MEK inhibitors are allosteric inhibitors that block MEK in its inactive form and have low efficacy when given as monotherapy due to hyperactivation of the upstream pathway [2].

Monotherapy of *BRAFV600E* mutant cell lines can increase EGFR phosphorylation, leading to adaptive feedback reactivation of the MAPK signaling pathway and continued cell proliferation, causing resistance to BRAF inhibitors. The addition of MEK inhibitors to BRAF inhibition could delay the development of this acquired resistance by blocking ERK signaling as well as prevent paradoxical MAPK pathway activation in the development of secondary squamous cell skin cancers, hence the development of combination treatments [3]. The mechanism of resistance by MAPK pathway inhibitors is further explored in Section 7.

## 4. BRAF/MEK Inhibitors across Various Cancers

### 4.1. Melanoma

*BRAF* mutations are found in approximately 40–60% of melanoma cases [4,9]. Vemurafenib was the first BRAF inhibitor to have FDA approval on 17 August 2011 for unresectable and metastatic melanoma, followed by dabrafenib on 29 May 2013 [10,11]. Vemurafenib was compared to the standard treatment, dacarbazine, in the phase III randomized BRIM-3 trial in patients with previously untreated *BRAFV600E* mutated metastatic melanoma [9]. Overall survival (OS) at 6 months was 84% in the vemurafenib group and 64% in the dacarbazine group [9]. The hazard ratio (HR) for death in the vemurafenib group was 0.37 (95% CI 0.26–0.55; *p* < 0.001) [9]. Progression-free survival (PFS) was 5.3 months in the vemurafenib group and 1.6 months in the dacarbazine group (HR 0.26, 95% CI 0.2–0.33; *p* < 0.001) [9]. Both coprimary end points, OS and PFS, were met in the study. The most common adverse effects of vemurafenib were cutaneous events, including rash in 71% of patients, squamous cell skin cancer in 20%, and keratoacanthoma in 11% of patients. Other common adverse effects were arthralgia, alopecia, fatigue, nausea, and diarrhea. Treatment discontinuations due to adverse events were 7% in the vemurafenib group and 2% in the dacarbazine group [9]. The final updated OS from BRIM-3 was reported in 2017 with an OS of 13.6 months in the vemurafenib group vs. 9.7 months in the dacarbazine group (HR 0.81, 95% CI 0.67–0.98; *p* = 0.03) [12]. Vemurafenib was also evaluated in patients with metastatic melanoma of the brain, with results reported in 2017 (Table 1) [13].

The approval for dabrafenib monotherapy was based on the phase III randomized BREAK-3 trial in treatment-naive patients with *BRAFV600E*-mutated unresectable or metastatic melanoma [14,15]. Median PFS was 6.7 months for dabrafenib and 2.9 months for dacarbazine (HR 0.35, 95% CI 0.20–0.61; *p* < 0.001). The most common adverse effects of dabrafenib were hyperkeratosis, headache, arthralgia, and pyrexia. Similar to vemurafenib, dabrafenib has skin-related toxic effects, including squamous cell skin carcinoma or keratoacanthoma [14,15]. Dose reductions were seen in 28% of patients on vemurafenib vs. 17% on dacarbazine, with drug discontinuations in 3% of patients in each group [14]. Updated results from the BREAK-3 trial in 2013 showed consistent PFS improvement, with a median PFS of 6.9 months and 2.7 months in the dabrafenib and dacarbazine groups, respectively (HR 0.37, 95% CI 0.23–0.57) [15]. However, OS was confounded by the crossover from dacarbazine to dabrafenib arm [15]. The phase II BREAK-MB trial evaluated 172 patients with BRAFV600-mutated melanoma with brain metastases. Patients were divided into cohort A with no prior therapy for brain metastasis and cohort B with prior local therapy for brain metastasis [16]. This study confirms that dabrafenib has efficacy in patients with *BRAFV600E* mutant melanoma and brain metastases, irrespective of prior local therapies [16] (Table 1).

Trametinib was the first MEK inhibitor to have FDA approval on 29 May 2014 for unresectable and metastatic melanoma based on the phase III randomized METRIC trial [17,18]. Updated results from the METRIC trial in 2019 showed a median PFS of 4.9 months and a median OS of 15.6 months in the trametinib arm vs. a median PFS of 1.5 months and a median OS of 11.3 months in the chemotherapy arm (PFS: HR 0.54, 95% CI 0.41–0.73 and OS: HR 0.84, 95% CI 0.63–1.11) [19]. The most common adverse events of trametinib were rash, diarrhea, fatigue, peripheral edema, nausea, and dermatitis acneiform [17].

Acquired resistance and development of secondary squamous cell skin cancers and other skin toxicities associated with BRAF inhibitors caused by the paradoxical activation of wild-type BRAF kinase in the MAPK pathway in normal tissues were the challenges faced with BRAF inhibitor monotherapy. Therefore, further investigations to overcome those resistances and toxicities led to the combination of BRAF and MEK inhibitors [20]. Dual BRAF and MEK inhibition improves efficacy and lessens the paradoxical MAPK activation-related toxicities [20]. Currently, two BRAF-MEK inhibitor combinations, dabrafenib–trametinib and vemurafenib–cobimetinib, were FDA-approved on 9 January 2014 and 10 November 2015, respectively, for the treatment of unresectable or metastatic BRAF mutant melanoma [21,22].

The approval for dabrafenib–trametinib was based on the phase III randomized COMBI-d trial of *BRAFV600E/K* mutated metastatic melanoma [20]. The dabrafenib–trametinib group has a longer median PFS than the group with dabrafenib only (9.3 months vs. 8.8 months, HR 0.75, 95% CI 0.57–0.99; *p* = 0.03) [20]. The efficacy was confirmed in the updated results in 2017 with a 3-year PFS of 22% in the dabrafenib–trametinib group vs. 12% with the dabrafenib monotherapy group (HR 0.71, 95% CI 0.57–0.88) and a 3-year OS of 44% versus 32%, respectively (HR 0.75, 95% CI 0.58–0.96) [23]. More dose modifications and more pyrexia, chills, diarrhea, and vomiting were noted in the combination group, but the rate of hyperkeratosis, alopecia, and cutaneous squamous cell carcinoma was lower in the combination group [20]. Dabrafenib–trametinib was also compared to vemurafenib monotherapy in the COMBI-v trial. The dabrafenib–trametinib group demonstrated better median OS (not reached) and median PFS (11.4 months) compared to OS of 17.2 months and PFS of 7.3 months in the vemurafenib monotherapy group (OS: HR 0.69, 95% CI 0.53–0.89; *p* = 0.005 and PFS: HR 0.56, 95% CI 0.46–0.69; *p* < 0.001) [24].

The dabrafenib–trametinib combination was also investigated in metastatic melanoma with brain metastasis in the phase II COMBI-MB trial, and it showed clinical efficacy, but the duration of response (DOR) was relatively short [25]. On the other hand, in the phase III COMBI-AD trial, combined dabrafenib–trametinib as an adjuvant treatment in completely resected melanoma with *BRAFV600E/K* mutation demonstrated a 3-year relapse-free survival rate (RFS) of 58% in the combined group vs. 39% in the placebo group (HR 0.47, 95% CI 0.39–0.58; *p* < 0.001) with a 3-year OS of 86% and 77%, respectively (HR 0.57; 95% CI, 0.42–0.79; *p* = 0.0006) [26]. This led to FDA approval of dabrafenib–trametinib as an adjuvant therapy in patients with *BRAFV600E/K* mutated resected melanoma on 30 April 2018 [27].

Another phase III randomized coBRIM trial led to the FDA approval of vemurafenib–cobimetinib in treatment-naive, *BRAFV600*-mutated metastatic melanoma patients [28,29]. It showed an improved median PFS and a median OS of 12.3 and 22.3 months in the combination group vs. 7.2 and 17.4 months, respectively, in the vemurafenib monotherapy group (PFS: HR 0.58, 95% CI 0.46–0.72; *p* < 0.0001 and OS: HR 0.70, 95% CI 0.55–0.90; *p* = 0.005) [28,29]. Updated results in 2021 maintained the efficacy of the vemurafenib–cobimetinib combination with a median OS and PFS benefit of 5.1 months and 5.4 months, respectively [30].

However, both combinations above are associated with disease progression at approximately 12 months and have unique toxic effects, prompting the development of more effective and better-tolerated treatments. This led to a new combination of encorafenib and binimetinib. Encorafenib, a new generation of ATP-competitive BRAF inhibitors, can have improved sustained target inhibition due to longer pharmacodynamic activity with a half-life of 10 times longer than either vemurafenib or dabrafenib [31]. The FDA approved the combination of encorafenib and binimetinib for the treatment of unresectable or metastatic melanoma patients with a *BRAFV600E* or *V600K* mutation on 27 June 2018 [32]. The approval was based on the phase III randomized active-controlled three-arm COLUMBUS trial, which evaluated the combined encorafenib–binimetinib vs. encorafenib alone or vemurafenib alone in patients with *BRAF*-mutated unresectable or metastatic melanoma [31]. Encorafenib–binimetinib demonstrated an improved median PFS of 14.9 months compared to 7.3 months in the vemurafenib group (HR 0.54, 95% CI 0.41–0.71; *p* < 0.0001) and 9.6 months in the encorafenib-only group (HR 0.75, 95% CI 0.56–1.0; *p* = 0.051) [31]. Encorafenib–binimetinib also showed OS benefits at 16.7 months compared to the vemurafenib group but no statistically significant OS improvement when compared to the encorafenib-only group [33]. Drug discontinuation rates due to adverse effects were similar in all three groups. The most common adverse effects in the combination group were nausea, diarrhea, vomiting, fatigue, arthralgia, and an increased serum creatinine phosphokinase level [33]. Overall, the combination of encorafenib and binimetinib showed a tolerable toxicity profile and could be used in patients who cannot tolerate other BRAF-MEK inhibitor combinations [31]. Five-year updated results of the COLUMBUS trial maintained the efficacy and durability of response in the combined encorafenib and binimetinib group with a PFS of 23% and an OS of 35%, while the PFS was 10% and the OS was 21% in the vemurafenib group [34].

**Table 1:** Landmark trials of BRAF/MEK-targeted monotherapy, combination therapy, and combinations with immune checkpoint inhibitors leading to FDA approvals and other important trials.

Study	Phase	Study Population	Number of Patients/ Subgroup	Intervention/Subgroup	PFS Months	OS Months	ORR (%)	DOR Months	FDA Approval/Notes
Chapman et al. BRIM-3 2011 [9,12]	III	Metastatic melanoma with *BRAFV600E* mutation, treatment-naive	337	vemurafenib	5.3	13.6	48	-	17 August 2011
			338	dacarbazine	1.6	9.7	5	-	
Ascierto et al. CoBRIM 2015 [28,29,30]	III	Unresectable or metastatic *BRAFV600* mutated melanoma, treatment-naive	247	vemurafenib + cobimetinib	12.3	22.3	70	13	10 November 2015
			248	vemurafenib + placebo	7.2	17.4	50	9.2	
McArthur et al. 2017 [13]	II	Metastatic *BRAFV600* mutant melanoma with brain metastasis (BM). Cohort A: previously untreated BM; Cohort B: previously treated BM	Cohort A 90	vemurafenib	3.7	8.9	33 EC 18 IC	4.1	EC: extracranial response IC: intracranial response
			Cohort B 56	vemurafenib	4	9.6	23 EC 18 IC	4.1	
Hauschild et al. BREAK-3 2012 [14,15]	III	Unresectable or metastatic melanoma with *BRAFV600E* mutation, treatment-naive	187	dabrafenib	6.9	18.2	50	5.5	29 May 2013
			63	dacarbazine	2.7	15.6	6	NR	
Long et al. BREAK-MB 2012 [16]	II	Unresectable or metastatic melanoma with *BRAFV600E* or *V600K* mutation, with brain metastasis Cohort A with no prior local therapy for brain metastasis and Cohort B with prior local therapy	Cohort A 83	dabrafenib V600E	16.1	33.1	39.2	20.1	29 May 2013 (ORR here is overall intracranial response)
				V600K	8.1	16.3	30.8	28.1	
			Cohort B 139	dabrafenib V600E	16.6	31.4	6.7	12.4	
				V600K	15.9	21.9	22.2	16.6	
Long et al. COMBI-d 2014 [20,23]	III	Unresectable or metastatic *BRAFV600E* or *V600K* mutant melanoma	211	dabrafenib + trametinib	9.3	NR	68	9.2	9 January 2014
			212	dabrafenib + placebo	8.8	NR	55	10.2	
Robert et al. COMBI-v 2014 [24]	III	Unresectable or metastatic melanoma with *BRAFV600E/K* mutation, treatment-naive	353	dabrafenib + trametinib	11.4	NR	64	13.8	
			353	vemurafenib	7.3	17.2	51	7.5	
Davies et al. COMBI-MB 2017 [25]	II	Metastatic melanoma with *BRAFV600E/K* mutation with brain metastasis. Cohort A: asymptomatic patients with *V600E* mutation and no prior local brain therapy. Cohort B: asymptomatic patients with *V600E* mutation who had prior local therapy. Cohort C: asymptomatic patients with *V600D/K/R* mutations regardless of prior local therapy. Cohort D: symptomatic patients regardless of local therapy or mutation status		dabrafenib + trametinib					
			76	Cohort A	5.6	10.8	58	6.5	
			16	Cohort B	7.2	24.3	56	12.5	
			16	Cohort C	4.2	10.1	44	6.6	
			17	Cohort D	5.5	11.5	65	4.5	
Long et al. COMBI-AD 2017 [26]		Stage III melanoma with completely resected *BRAFV600E/K*-mutated tumor	438	dabrafenib + trametinib	NR	NR	37 (recurrence)		30 April 2018
			432	placebo	NR	NR	56 (recurrence)		
Flaherty et al. METRIC 2012 [17,19]	III	Unresectable or metastatic melanoma with *BRAFV600E* or *V600K* mutation, treatment-naive and previously treated	214	trametinib	4.9	15.6	29	5.3	29 May 2013
			108	dacarbazine or paclitaxel	1.5	11.3	9	8.1	
Dummer et al. COLUMBUS 2018 [31,33,34]	III	Unresectable or metastatic melanoma, treatment-naive or progressed after first-line immunotherapy	192	encorafenib + binimetinib	14.9	33.6	63	18.6	27 June 2018
			194	encorafenib	9.6	23.5	51	14.9	
			191	vemurafenib	7.3	16.9	40	12.3	
Salama et al. NCI-MATCH subprotocol H 2020 [35]	II	Previously treated *BRAFV600E*-mutated tumors, excluding melanoma, thyroid, and colorectal cancer. Responses seen in 7 distinct tumor types	35	dabrafenib + trametinib	11.4	28.6	38	25.1	23 June 2022 tumor-agnostic indication for solid tumors
Kopetz et al. BEACON CRC 2019 [36,37]	III	*BRAFV600E*-mutated mCRC, treatment-naive	220	encorafenib + cetuximab	4.3	9.3	20	-	8 April 2020 for encorafenib and cetuximab for mCRC
			224	encorafenib + cetuximab + binimetinib	4.5	9.3	26	-	
			221	cetuximab + irinotecan or cetuximab + FOLFIRI	1.5	5.9	2	-	
Cutsem et al. ANCHOR CRC 2023 [38]	II	*BRAFV600E*-mutated mCRC, treatment-naive	95	encorafenib+ binimetinib+ cetuximab	5.8	18.3	47.4	-	
VE-BASKET 2015 [39,40,41,42,43]		*BRAFV600*-mutated nonmelanoma cancers (26 unique cancer types)	Total 172	vemurafenib	5.8	17.6	32.6	13.1	vemurafenib for ECD on 6 November 2017
			NSCLC (62)	vemurafenib	6.5	15.4	37.1	7.2	
			ECD (22)/LCH (4)	vemurafenib	NR	NR	61.5	-	
			Glioma (24)	vemurafenib	5.5	28.2	25	-	
			CRC (27)	vemurafenib	4.5	9.3	0	-	
				vemurafenib + cetuximab	3.7	7.1	4	-	
			ATC (7)	vemurafenib	-	-	29	-	
			BTC (26)	vemurafenib	-	-	12	-	
Subbiah et al. ROAR Basket trial 2023 [44,45]	II	*BRAFV600E* mutated rare cancers, ATC, BTC, ASi, LGG, HGG, HCL, MM	ATC (36)	dabrafenib + trametinib	6.7	14.5	56	14.4	4 May 2018 for ATC 23 June 2022 tumor-agnostic indication for solid tumors
			BTC (43)	dabrafenib + trametinib	9	13.5	53	8.9	
			ASi (3)	dabrafenib + trametinib	-	21.8	67	7.7	
			LGG (13)	dabrafenib + trametinib	9.5	NR	54	NR	
			HGG(45)	dabrafenib + trametinib	5.5	17.6	33	31.2	
			HCL (55)	dabrafenib + trametinib	NR	NR	89	NR	
			MM (10)	dabrafenib + trametinib	6.3	33.9	50	11.1	
Planchard et al. BRF113928 2016–2017 Updated 2022 [46,47,48]	II	*BRAFV600E*-mutated metastatic NSCLC. Cohorts A and B: previously treated and Cohort C: treatment-naive	Cohort A (78)	dabrafenib	5.5	12.6	33	9.6	22 June 2017 for dabrafenib and trametinib
			Cohort B (57)	dabrafenib + trametinib	10.2	18.2	68.4	9.8	
			Cohort C (36)	dabrafenib + trametinib	10.8	17.3	63.9	10.2	
Mazieres et al. French AcSe 2020 [49]	II	*BRAF*-mutated NSCLC cohort, previously treated. Cohort A: *BRAF* non*V600* mutation. Cohort B: *BRAFV600* mutations	Cohort A (15)	vemurafenib	2.1–6.8	-	0	-	
			Cohort B (100)	vemurafenib	5.2	10	44.8	6.4	
Riely et al. PHAROS trial 2023 [50]	II	*BRAFV600E*-mutated metastatic NSCLC. Cohort A: treatment-naive and Cohort B: previously treated	Cohort A (59)	encorafenib + binimetinib	NR	NR	75	NR	12 October 2023
			Cohort B (39)	encorafenib + binimetinib	9.3	NR	46	16.7	
Diamond et al. 2022 [51]	II	ECD/LCH/RDD patients regardless of *BRAF* mutations	18	cobimetinib	NR	-	89	NR	1 November 2022
Whitlock et al. CDRB436A2102 2023 [52]	I/II	R/R *BRAFV600*-mutated pediatric LCH	13	dabrafenib	NR	-	76.9	NR	
Whiltlock et al. CTMT212X2101 2023 [52]	I/II	R/R *BRAFV600*-mutated pediatric LCH	12	dabrafenib + trametinib	NR	-	58.3	NR	
Gershenson et al. 2022 [53]	II/ III	Recurrent LGSOC	130	trametinib	13	37.6	26	13.6	
			130	SOC	7.2	29.2	6	5.9	
Ribas et al. KEYNOTE-022 2020 Updated 2022 [54]	I/II	*BRAFV600E/K*-mutated melanoma, treatment-naive	60	pembrolizumab + dabrafenib + trametinib	17	46.3	65	30.2	
			60	dabrafenib + trametinib	9.9	26.3	72	12.1	
Gutzmer et al. IMspire150 2020 Updated 2023 [55]	III	Advanced or metastatic *BRAFV600* mutant melanoma	256	atezolizumab + vemurafenib + cobimetinib	15.1	39.0	67	21	30 July 2020
			258	vemurafenib + cobimetinib	10.6	25.8	65	12.6	

ASi: adenocarcinoma of the small intestine, ATC: anaplastic thyroid carcinoma, BTC: biliary tract cancer, CRC: colorectal cancer, ECD: Erdheim–Chester disease, GIST: gastrointestinal stromal tumor, HCL: hairy cell leukemia, HGG: high-grade glioma, LCH: Langerhans cell histiocytosis, LLG: low-grade glioma, and MM: multiple myeloma. NR—not reached and (-)—data not available.

### 4.2. Gastrointestinal Cancers

#### 4.2.1. Colorectal Cancer

*BRAF* mutations are found in approximately 10% of metastatic colorectal cancer (mCRC) and up to 20% of colorectal cancer overall [36,56]. Non-*V600E* mutations represent about 2.2% of mCRC [56]. Somatic BRAF mutations increase the BRAF signaling pathway, resulting in CpG island methylation, which silences the tumor suppressor gene MLH1 and leads to deficient DNA mismatch repair. Therefore, microsatellite instability (MSI) can be found in 20% of mCRC patients with *BRAFV600E* mutations [56]. In sporadic CRC, 60% of MSI-high tumors can have a *BRAF* mutation [56]. The presence of the *BRAFV600E* mutation is a marker for poor prognosis before the utilization of targeted therapies and is associated with older age at diagnosis, female sex, right-sidedness, nodal and peritoneal metastasis, poorer differentiation, mucinous histology, larger primary tumors, and *KRAS* wild-type tumors [56]. Non-*V600E BRAF* mutations are more likely to be associated with younger patients, male sex, well-to-moderately differentiated and left-sided primary tumors, concomitant *RAS* mutations, and lower MSI [56]. Non-*BRAFV600E* mutations do not have a negative impact on prognosis and present with a longer OS compared to *BRAFV600* mutant CRC or wild-type CRC [56].

Dual blockade of both EGFR and BRAF has been shown to have synergistic inhibition in *BRAFV600E* mutant colorectal cancer murine models [56]. The landmark phase III BEACON CRC trial led to FDA approval of the encorafenib and cetuximab combination in previously treated mCRC with the *BRAFV600E* mutation on 8 April 2020 [57]. BEACON CRC divided patients into three groups, as summarized in Table 1. Encorafenib and cetuximab groups have a better median OS of 8.4 months compared to 5.4 months in the control group (irinotecan with cetuximab or FOLFIRI (5 fluorouracil and irinotecan) with cetuximab) (HR 0.60, 95% CI 0.45–0.79; *p* = 0.0003), with an improved ORR of 20% compared with 2% in the control group (*p* < 0.001) [36]. The trial was not powered enough to compare triplet and doublet combinations, and the triplet therapy (encorafenib, binimetinib, and cetuximab) was not FDA-approved yet due to the comparable clinical outcomes with doublet therapy [36]. However, triplet therapy has manageable toxicities, thus paving the way for further investigations of its utilization in mCRC [58]. The most common grade 3 or 4 adverse effects were fatigue, elevated aspartate transferase (AST) level, and urinary tract infections; in the most common grade, any adverse effects were diarrhea, dermatitis acneiform, fatigue, nausea, dry skin, and drug discontinuations of at least one drug due to adverse events were seen in 20% of patients [36]. Updated results of BEACON-CRC were reported in 2021, as summarized in Table 1 [37].

The phase II ANCHOR-CRC trial evaluated the combination of encorafenib, binimetinib, and cetuximab in the first-line setting in *BRAFV600E*-mutated mCRC. The primary end point ORR was met with an ORR of 47.4%, a median PFS of 5.8 months, and a median OS of 18.3 months [38]. The most frequent grade 3 or higher adverse events were anemia, diarrhea, nausea, and large-intestine obstruction [38]. Currently, encorafenib and cetuximab are being evaluated in combination with chemotherapy as a first-line treatment in *BRAFV600E*-mutated mCRC in the phase III BREAKWATER trial (NCT04607421) [59].

#### 4.2.2. Biliary Tract Cancers

BRAFV600E mutations are seen in approximately 4–7% of biliary tract cancers (BTC) and are observed more in the intrahepatic BTC [60,61]. Intrahepatic BTC patients with BRAFV600E mutations tend to have a higher tumor stage at surgery, a higher lymph node involvement, and an overall worse prognosis than the non-*BRAF*-mutated BTC patients [62]. Updated results of the *BRAFV600E*-mutated BTC cohort from the phase II ROAR trial reported in 2023 showed that patients treated with dabrafenib and trametinib had an ORR of 53%, a DOR of 8.9 months, and a median PFS of 9 months [44]. The phase II NCI MATCH EAY131-H trial, including four patients with *BRAFV600E*-mutated intrahepatic BTC, confirmed an ORR of 38% with a median PFS of 11.4 months in the overall population. Among them, 75% of BTC patients had partial responses (PR) [35]. There is no specific indication of MAPK pathway inhibitors in BTC, but based on those trials, dabrafenib and trametinib were approved by the FDA on 23 June 2022 for a tumor-agnostic indication for unresectable or metastatic *BRAFV600E* mutated solid tumors that have progressed on prior therapy and have no other alternative treatment options [63].

### 4.3. Non-Small Cell Lung Cancer

*BRAF* mutations are found in approximately 3–5% of non-small cell lung cancer (NSCLC) patients, while *BRAFV600E*-specific mutations are only present in about 2% of NSCLC [2,64]. *BRAFV600E* mutations in NSCLC are more common in micropapillary patterns and females with no smoking history, while non-*V600E BRAF* mutations are more likely associated with mucinous patterns and males with a smoking history [2]. *BRAF* mutations are associated with metastasis to the central nervous system (CNS), especially with class II and III mutations [65]. In an analysis of *BRAF*-altered samples by Negrao et al., the most common BRAF mutations were missense mutations (90%, with 45% of which were variants of unknown significance), followed by nonsense and splice-site mutations (5% each) [64]. Class I mutations are exclusively *V600E* mutations, whereas *G469A* and *K601E* are the most common class II mutations, and *G466V* and *N581S* are the most common class III mutations [64]. The most common co-mutations in *BRAF* that altered NSCLC were *TP53* (57%), *EGFR* (26%), *KRAS* (15%), and *NF1* (15%) [64]. All class *BRAF* mutations co-occurred in 10% of samples with EGFR activating exon 21 *L858R* and exon 19 deletion mutations, whereas class III BRAF mutations are more likely to have *KRAS* mutations than class I and II mutations (I: 6.0%; II: 12.6%; III: 23.5%; and *p* < 0.01) [64]. *BRAF* mutations also seem to be associated with PDL1 (programmed death ligand 1) expressions, with more than 50% of PDL1 expressions reported in 42% of *BRAFV600E* and 50% of non-*V600E* mutations [66]. On the other hand, *BRAF* fusions were identified in 0.2% of 17,128 NSCLC samples, with the most frequent partner genes being *AGK*, *DOCK4*, and *TRIM24*, and the most frequently co-occurring mutations being *TP53* (67%), *CDKN2A* (31%), *EGFR* (29%), and *CDKN2B* (26%) [67]. *MEK* alterations are very rare, with only 0.6% present in 6024 lung adenocarcinoma cases, associated with current smoking status [68]. The prognostic value of *BRAFV600E* in NSCLC is unclear, but patients with this mutation seem to have poorer outcomes and a lower response to platinum-based chemotherapy [48].

In the French Acsé phase II trial, vemurafenib was evaluated in two cohorts of NSCLC patients with either *BRAFV600E* or non-*V600 BRAF* mutations [49]. ORR was 0% in the non-*V600* cohort, and ORR was 44.8% in the *BRAFV600E* cohort, with a DOR of 6.4 months, a median PFS of 5.2 months, and a median OS of 10 months [49].

The combination of dabrafenib and trametinib was FDA-approved on 22 June 2017 for the treatment of NSCLC harboring *BRAFV600E* mutations as both the first and second lines [69]. The approval was based on the phase II BRF113928 trial, which divided *BRAFV600E*-mutated metastatic NSCLC patients into three cohorts. Patients in cohorts A and B received at least one prior therapy, while patients in cohort C were treatment-naive [47,48]. Cohort A received dabrafenib alone, and cohorts B and C received both dabrafenib and trametinib. Patients in cohort A have an ORR of 33% with a median PFS of 5.5 months, while patients in cohort B have an ORR of 63.2% with a median PFS of 9.7 months [47]. ORR of 64% was observed in cohort C with a DOR of 10.4 months and a median PFS of 10.9 months [48]. Updated results from the BRF113928 trial were reported in 2022, with results listed in Table 1, which continued to maintain the efficacy of combined dabrafenib and trametinib in the *BRAFV600E*-mutated NSCLC regardless of prior treatments [46]. However, the dabrafenib–trametinib combination can lead to increased adverse effects, given the added toxicities of each drug. In a meta-analysis of the toxicities of BRAF and MEK inhibitors by Garutti et al., 95% of patients who received both dabrafenib and trametinib had all grade adverse events, with grade 3 or higher adverse events seen in 43% of patients, with the most common being pyrexia, rash, and hypertension [70]. Dose reductions were seen in 28% of patients with dabrafenib and trametinib, while drug discontinuations were seen in 24% of patients [70]. Due to the significant adverse effects of dabrafenib–trametinib and its low efficacy, the dabrafenib–trametinib combination is usually used as a second line after the chemoimmunotherapy combination with pembrolizumab in metastatic NSCLC with BRAF mutations, unlike other tyrosine kinase inhibitors, which are used in the first-line setting for specific molecular alterations.

The combination of encorafenib and binimetinib in both treatment-naive and previously treated BRAF-mutated NSCLC is currently being evaluated in two phase II trials, the PHAROS (NCT03915951) and ENCO-BRAF trials. The interim analysis of the PHAROS trial showed an ORR of 75% in the treatment-naive group and 46% in previously treated patients, with more detailed results summarized in Table 1 [50]. The most frequent adverse events were nausea, diarrhea, and fatigue, with 24% of dose reductions and 15% of drug discontinuations seen [50]. This led to the FDA’s approval of encorafenib and binimetinib for the treatment of adult patients with metastatic NSCLC with the BRAFV600E mutation on 12 October 2023 [71].

### 4.4. Hematological Malignancies

#### 4.4.1. Hairy Cell Leukemia

Tiacci et al. reported in 2011 that the *BRAFV600E* mutation is the disease-defining genetic alteration in hairy cell leukemia (HCL) and absent in other B cell leukemias and lymphomas and plays a pivotal role in HCL cell survival [72,73]. The *BRAFV600E* mutation occurs in 90–100% of HCL cases and is both a diagnostic and therapeutic target in HCL [45,72,74]. A variant type of HCL (HCL-V) has no *BRAFV600E* mutation but is instead found to have *MEK1* (*MAP2K1*) mutations in half of the cases [75].

In two different phase II trials in Italy and the United States, vemurafenib showed a high ORR of 96–100% when administered for a median of 16–18 weeks in relapsed and refractory (R/R) HCL, but the median RFS was short at 9 months [76]. However, it was significantly longer in patients with complete responses (CR) than in patients with PR (19 months vs. 6 months, HR 0.26, 95% CI 0.1–0.68, *p* = 0.006) [77]. Vemurafenib given in combination with rituximab for 8 weeks in a phase II trial showed an ORR of 87% (all CR, *p* = 0.005) with improved DOR [77]. Median RFS was not reached at a median follow-up of 19.5 months compared to the shorter DOR observed with BRAF inhibitor monotherapy [77]. Median RFS was even longer in patients with MRD negativity [77].

In the phase II ROAR basket trial evaluating dabrafenib and trametinib in *BRAFV600E* mutated rare tumors, a cohort of R/R HCL was reported to have an ORR of 89% and an MRD negativity of 12.7%, making it a potential therapeutic option for the R/R *BRAFV600E* mutant HCL [44,45].

#### 4.4.2. Langerhans Cell Histiocytosis/Erdheim–Chester Disease

Langerhans Cell Histiocytosis (LCH) is a rare clonal neoplasm derived from macrophage and dendritic lineages primarily occurring in children, with more than 50% of patients having *BRAF* mutations [42,52]. It is characterized by the uncontrolled multiplication and accumulation of cells similar to Langerhans cells in bones, skin, and visceral organs such as the liver and lungs [52]. *BRAF* mutations have been associated with a more severe disease, a poorer prognosis, and a higher prevalence in younger patients [52]. On the other hand, Erdheim–Chester Disease (ECD) and Rosai–Dorfman Disease (RDD) are non-LCH with multiorgan involvement, including diffuse osteosclerotic lesions, orbital infiltration, lung, kidney, cardiac, and neurological involvement, as well as other endocrinopathies [78]. *BRAFV600E* mutations are found in half of patients with ECD, while patients without *BRAFV600E* mutations tend to have other mutations in components of the MAPK pathway, including the *RAS* and *MEK1* genes [78].

The phase II VE-BASKET trial evaluated vemurafenib in 22 patients with *BRAFV600*-mutated ECD and four patients with LCH, including treatment-naive patients [42]. ORR was 61.5% in the overall cohort and 54.5% in patients with ECD, while PFS and OS were not reached at a median follow-up of 28.8 months at study closure [42]. Two-year OS and PFS were 83% and 95%, respectively, in the ECD patients [42]. This led to FDA approval of vemurafenib for ECD on 6 November 2017 [79].

Given that almost all patients with histiocytosis have either *BRAFV600* mutations or some type of molecular alterations in the MAPK pathway, further research has been developed for the use of MEK inhibitors [51]. Cobimetinib was evaluated in ECD/LCH patients regardless of *BRAF* mutation status and was found to have an ORR of 89% with a DOR and PFS not reached at a median follow-up of 11.9 months [51]. Responses were observed in patients with *ARAF*, *BRAF*, *RAF1*, *NRAS*, *KRAS*, *MEK1*, and *MEK2* mutations [51]. Based on those results, the FDA approved cobimetinib for adult patients with histiocytic neoplasms, including ECD, RDD, and LCH, on 1 November 2022 [80].

Dabrafenib monotherapy in CDRB436A2102 and in combination with trametinib in CTMT212X2101 demonstrated clinical efficacy and manageable toxicity in pediatric patients with the R/R *BRAFV600* mutant LCH [52]. ORR was 76.9% in the dabrafenib monotherapy arm with both a 12- and 24-month DOR of 90%, while ORR was 58.3% in the dabrafenib plus trametinib trial with a 12- and 24-month DOR of 100% [52].

In a retrospective study evaluating the benefit of trametinib in patients with ECD, 35% of patients had *BRAFV600E* mutations, and ORR was seen in 71% of patients, while OS and PFS were not reached at a median follow-up of 23 months [78]. Out of the responders, 73% of patients did not have the *BRAFV600E* mutation but instead had other alterations in the MAPK pathway, including *MEK1* or *RAS* [78]. In addition, given that the time-to-treatment failure was 37 months, ECD patients treated with trametinib have more durable responses and do not seem to have developed the acquired resistance like in other solid tumors [78]. However, both dabrafenib and trametinib did not have specific FDA approval for histiocytosis neoplasms.

### 4.5. Central Nervous System Tumors

*BRAF* mutations, including *BRAFV600E*, are present in about 7% of all CNS tumors, with prevalence in 60% of pleomorphic xanthoastrocytomas (PXA), 10–12% of anaplastic PXA, 80–95% of benign papillary craniopharyngioma, 38% of astroblastoma, 20–70% of gangliogliomas, 10% of pilocytic astrocytoma, and 1–2% of adult glioblastomas (GBM) [4,81]. Class I *BRAF* mutations represent 44–66% of all *BRAF* mutations in gliomas, while class II and III mutations represent 10–24% and 4–10%, respectively [81]. GBMs with *BRAF* mutations are different in different aspects, including location, survival rates, and global gene-expression profiles, from the rest of the GBMs. GBM patients with *BRAF* mutations are usually younger, with a longer survival rate when compared with other patients with GBM and epithelioid features [7]. The *BRAFV600E* mutation is more common in IDH-wild-type tumors (GBM) than in IDH-mutant tumors, which are now classified as astrocytomas [81]. In diffuse low-grade gliomas, the *BRAFV600E* mutations are found in 2–5% of cases [81]. However, the impact of the *BRAFV600E* mutation on the prognosis of gliomas is unclear [82]. Multiple case reports have shown some clinical efficacy of BRAF inhibitors (vemurafenib, dabrafenib) either alone or in combination with MEK inhibitors (trametinib) in *BRAF*-mutated glioma patients [4].

Vemurafenib was evaluated in a phase II VE-BASKET trial of non-melanoma *BRAFV600E* mutant tumors, which included 24 patients with different glioma subtypes [40]. The glioma cohort enrolled patients with a malignant diffuse glioma (GBM and anaplastic astrocytoma), PXA, an anaplastic ganglioglioma, a pilocytic astrocytoma, and a high-grade glioma, not otherwise specified [40]. ORR was 25% with a median PFS of 5.5 months and a median OS of 28.2 in the overall population [40]. Malignant diffuse gliomas have an ORR of 9.1% with a median PFS of 5.3 months and a median OS of 11.9 months [40].

Subsequently, the dabrafenib and trametinib combination was also evaluated in another phase II ROAR basket trial, which included *BRAFV600E*-mutated rare tumors [44]. The study included high-grade glioma (HGG) and low-grade glioma (LGG) cohorts [82]. ORR in LGG was 54% with one CR and six PR, while ORR in HGG was 33% with three CR and twelve PR with good DOR (Table 1) [44]. The dabrafenib–trametinib combination seems to have a better ORR than vemurafenib alone.

Based on the ROAR trial, dabrafenib and trametinib were approved by the FDA on 23 June 2022, as tumor-agnostic indications in patients with unresectable or metastatic *BRAFV600E* solid tumors who have progressed on prior therapy and have no other alternative treatment options [63].

### 4.6. Thyroid Cancers

The *BRAFV600E* mutation is the most important and common genetic alteration in thyroid cancers, comprising 37–60% of papillary thyroid carcinoma (PTC) and 20–45% of anaplastic thyroid carcinoma (ATC) [1,83]. The presence of the *BRAF* mutation is associated with more aggressive tumor features, including extrathyroidal extension, advanced tumor stage at presentation, and metastasis in PTC [83].

Radioactive iodine therapy (RAI) is the main treatment for metastatic dedifferentiated thyroid cancer (DTC), of which 80% are PTC. However, two-thirds of those patients became refractory to RAI [84]. BRAF mutations can lead to hyperactivation of the MAPK pathway, which decreases the expression of the sodium/iodine symporter and reduces iodine uptake. Thus, MAPK pathway inhibitors also have the potential for re-sensitization to RAI by increasing iodine uptake via MAPK pathway inhibition [84]. The phase II MERAIODE trial showed that a combination of dabrafenib and trametinib is associated with reinduction of RAI in 95% of patients, with a 6-month response rate of 38% in patients with *BRAFV600E* and *RAS*-mutated, RAI-refractory DTC [85,86].

Vemurafenib was evaluated in the phase II trial of patients with *BRAFV600E*-positive PTC and demonstrated some efficacy [83]. The patients were divided into two cohorts. Cohort 1 included patients who never had a vascular endothelial growth factor (VEGFR) multi-kinase inhibitor, and cohort 2 included patients with prior VEGFR multi-kinase inhibitors [83]. Cohort 1 showed an ORR of 38% with a DOR of 16.5 months, while cohort 2 showed an ORR of 27.3% with a DOR of 7.4 months [83].

The combination of dabrafenib and trametinib has also shown efficacy in ATC in the phase II ROAR basket trial, which led to its FDA approval on 4 May 2018, for locally advanced or metastatic *BRAFV600E* mutated ATC with no satisfactory locoregional treatment options [87]. The ATC cohort of the ROAR trial included 16 patients with *BRAFV600E*-mutated ATC who had received prior radiation therapy/surgery, of which 6 had received prior systemic therapy. ORR was seen in 69% of patients [88]. Updated results in 2023 showed an ORR of 56%, with a DOR of 14.4 months, a median PFS of 6.7 months, and a median OS of 14.5 months, confirming its efficacy [44].

### 4.7. Gynecological Cancers

Low-grade serous ovarian carcinoma (LGSOC) is a subtype of ovarian carcinoma that accounts for 5–10% of all epithelial ovarian cancers. It has an indolent nature, low response rates to chemotherapy, and a high prevalence of MAPK pathway alterations [89]. There is a wide range of *BRAF* mutations in LGSOC depending on individual studies, but *BRAF* mutations are found in approximately 2–16% of LGSOC [89]. In the AACR GENIE cohort, *BRAF* mutations are found in 9.5% of LGSOC [90].

The phase III MILO/ENGOT-ov11 trial of binimetinib in LGSOC was closed prematurely after an interim analysis of the initial 303 patients showed futility with an ORR of 16%. PFS was 9.1 months in the binimetinib group and 10.6 months in the group with the physician’s choice chemotherapy (HR, 1.21; 95% CI 0.79–1.86) [91]. Even though its primary end point, PFS, was not met, it showed that MEK inhibition is beneficial in the disease control of some patients [91]. A subsequent molecular analysis report showed that the ORR of binimetinib was higher in patients with MAPK pathway alterations (41%) compared to patients without MAPK pathway alterations (13%) [89]. The most common MAPK alterations included *KRAS* and *BRAFV600E*, with the rest being *NRAS*, *RAF1*, and *NF1* alterations [89]. Therefore, binimetinib could be considered as an option for the treatment of recurrent LGSOC.

The phase II EAY131-H NCI-MATCH trial included five patients with LGSOC and one patient with mucinous papillary serous adenocarcinoma of the peritoneum [35]. All six patients had clinical benefits from dabrafenib and trametinib therapy, with PR in five patients and stable disease in one patient [35]. Results from the NCI MATCH trial, together with the ROAR trial, led to the FDA approval of dabrafenib and trametinib for *BRAFV600E*-mutated solid tumors, including gynecological cancers as tumor-agnostic indications [63].

Trametinib monotherapy in recurrent LGSOC in the GOG281/LOGS phase II/III trial demonstrated improved PFS of 13.0 months in the trametinib group compared to 7.2 months in the standard of care (SOC) group (HR 0.48, 95% CI 0.36–0.64; *p* < 0.0001) [53]. ORR was 26% in the trametinib group compared to 6% in the SOC (odds ratio 5.4, 95% CI 2.4–12.2, *p* < 0.0001) [53]. The study included patients regardless of their MAPK alteration status [53]. Thus, trametinib monotherapy can be considered as an option for LGSOC regardless of *BRAF* mutations.

## 5. Combinations of BRAF/MEK Inhibitors with Immunotherapy

Given the limitations seen in both immune checkpoint inhibitors (ICIs) and targeted therapies, combination therapy of ICIs with BRAF/MEK inhibitors has been further developed. The KEYNOTE-022 phase I/II trial evaluated the addition of pembrolizumab to dabrafenib and trametinib in patients with unresectable or metastatic melanoma in parts 1–3 and solid tumors in parts 4–5 [54]. Reports from the long-term follow-up of melanoma patients with *BRAFV600E/K* mutations were presented in 2022 [54]. Part 3 patients were randomized 1:1 to triplet therapy with pembrolizumab, dabrafenib, and trametinib and doublet therapy with dabrafenib and trametinib [54]. It reported a median PFS of 17.0 months for the triplet arm vs. 9.9 months for the doublet arm (HR 0.46, 95% CI 0.29–0.74). The median OS was 46.3 months in the triple arm and 26.3 months in the doublet arm [54]. DOR was higher in the triplet arm, with 30.2 months compared to 12.1 months in the doublet arm [54]. Another phase II TRICOTEL trial evaluated the same combination in previously untreated metastatic melanoma patients with brain metastasis of 5 mm or larger in at least one dimension [92]. Patients were divided into two cohorts: the *BRAFV600* mutant-positive cohort and the *BRAFV600* wild-type cohort [92]. Patients in the *BRAF* wild-type group had atezolizumab and cobimetinib, while patients in the *BRAFV600* mutant group had atezolizumab, vemurafenib, and cobimetinib [92]. The intracranial response rate was 42% by independent review charter (IRC) in the *BRAFV600* mutant cohort and 27% by investigator review in the *BRAFV600* wild-type cohort, showing some intracranial activity of triplet combination in *BRAF600* mutant melanoma with brain metastasis [92].

On the other hand, phase III IMspire150 evaluated atezolizumab in combination with vemurafenib and cobimetinib in *BRAFV600* mutants with advanced or metastatic melanoma. Primary analysis reported an improved median PFS of 15.1 months in the triplet arm vs. 10.6 months in the doublet arm with vemurafenib and cobimetinib (HR 0.78; 95% CI 0.63–0.97; *p* = 0.025) [50]. It led to FDA approval of a triplet combination as the first line in *BRAFV600* mutant unresectable or metastatic melanoma on 30 July 2020 [93]. Results from the second interim analysis were reported in 2023, and they showed better OS in the triplet arm, but the results were not statistically significant. The median OS was 39 months in the triplet arm vs. 25.8 months in the doublet arm (HR 0.84, 95% CI 0.66–1.06; *p* = 0.14); however, it continued to show PFS benefit and longer DOR with 21 months in the triplet arm vs. 12.6 months (95% CI 10.5–16.7) in the doublet arm [55]. However, another PDL1 antibody, spartalizumab, in combination with dabrafenib and trametinib in the phase III COMBI-I trial did not meet its primary end point PFS with a PFS of 16.2 months in the triplet arm vs. 12.0 months in the doublet arm (HR 0.82, 95% CI 0.66–1.03; *p* = 0.042 (one-sided; nonsignificant)) [94].

Despite better efficacy and more durable responses, there is concern that the combined use of BRAF/MEK inhibitors with ICIs could increase the overall adverse effects and intolerability. Therefore, there has been slow progress in the investigations of triplet combinations of ICIs and BRAF/MEK inhibitors. In the KEYNOTE-22 trial, 58% of patients in the triplet arm (dabrafenib, trametinib, and pembrolizumab) and 25% of patients in the doublet arm (dabrafenib and trametinib) had grade 3 or higher adverse events, with all grade treatment-related adverse events in 95% vs. 93%, dose interruptions in 83% vs. 68%, dose reductions in 27% vs. 15%, and discontinuations in 47% vs. 20% in the triplet arm vs. the doublet arm, respectively [95]. Immune-mediated adverse events occurred in 52% of patients in the triplet arm vs. 15% in the doublet arm, with the most common being pneumonitis, leading to one’s death and hypothyroidism [95]. More grade 3 or higher adverse events were also reported with the triplet arm (spartalizumab, dabrafenib, and trametinib) compared to the doublet arm (dabrafenib and trametinib) in the COMBI-I trial, with 55% of patients in the triplet arm vs. 33% in the doublet arm. More drug discontinuations (36% vs. 18%) and dose reductions (68% vs. 45%) were also reported in the triplet arm vs. the doublet arm [94]. However, treatment-related adverse effects of a triplet combination (vemurafenib, cobimetinib, and atezolizumab) were comparable to a doublet regimen (vemurafenib and cobimetinib) in the IMspire150 trial, as 99% of patients in both arms had adverse effects, with only 6% more of grade 3 or higher adverse events in the triplet arm (79% vs. 73%) [50]. Adverse events, including increased blood creatinine phosphokinase level, arthralgia, pyrexia, myalgias, increased liver enzymes, hyperthyroidism, hypothyroidism, and pneumonitis, were higher in the triplet arm compared to the doublet arm, while the incidence of a rash is similar in both groups. More drug discontinuations were, however, seen in the doublet group compared to the triplet group (16% vs. 13%) [50].

Currently, the encorafenib–binimetinib–pembrolizumab combination is being evaluated as a first line in *BRAFV600*-mutated melanoma in the STEABOARD phase III trial, which started enrollment in June 2022 [96]. Hopefully, this combination of ICI and BRAF/MEK inhibitors has more tolerable toxicities than prior combinations.

## 6. Mechanism of Resistance to BRAF/MEK Inhibitors

Resistance to anti-neoplastic therapy is a significant barrier to achieving long-term remission and disease control in cancer. Primary resistance occurs due to the initial lack of response to treatment, while secondary resistance occurs after the initial response to treatment. Targeted treatment with BRAF/MEK inhibitors is no exception to this. As discussed previously, the median PFS with single-agent BRAF inhibitors in BRAF-mutated metastatic melanoma is around 5–6 months [9,15]. The addition of MEK inhibitors improves this to about a year [24]. However, a subset of tumor cells acquires a resistance mechanism that allows them to evade targeted therapy and leads to disease progression. These resistance mechanisms represent a broad and complex interplay of tumor cell heterogeneity, tumor microenvironment, genetic and epigenetic changes, and reprogramming of metabolic pathways. They have been studied extensively in the melanoma population. It is evident that MAPK pathway activation is a key pathway in carcinogenesis and is targeted by BRAF and MEK inhibitors. Hence, most resistance mechanisms involve an alteration of the MAPK pathway that leads to its reactivation. The phosphorylation and activation of ERK1 and ERK2 is an important downstream step that is regulated and provides negative feedback to other signaling molecules such as SOS, SPRY, and DUSP proteins. The loss of this negative feedback loop can allow a subset of tumor cells to survive in the drug environment, with additional mutations driving further growth of tumor cells [97].

In a compilation of 132 tissue samples from three studies of resistance to *BRAF* inhibitors, the most commonly identified resistance mechanisms were *NRAS* or *KRAS* mutations (20%). Other common mechanisms included *BRAF* splice variants, *BRAF* amplification, *MEK* 1/2 mutations, and alteration in non-MAPK pathways, in order of their frequency. *NRAS* mutations were also associated with intracranial metastasis [98]. Overproduction of *BRAFV600E* due to amplification of the mutant gene is a common mechanism of resistance and leads to overactivation of the MEK pathway, causing resistance to both BRAF and MEK inhibitors. There are also four splicing site variants of *BRAFV600E* described that lack the RAS binding domain and can dimerize even with low levels of RAS. In patients with melanoma, up to 13–30% of cases are described as having acquired resistance due to abnormal splicing [97]. In an analysis by Van Allen et al., whole exome sequencing was performed in 45 patients with *BRAFV600E* mutated metastatic melanoma who received vemurafenib or dabrafenib monotherapy. Genomic alterations in known resistance genes were observed in 23 of 45 patients (51%) [99]. These were broadly divided into early treatment failures (less than 12 weeks) that are purported to have an inherent resistance mechanism and late progression, where an acquired resistance is developed. Alterations conferring resistance occurred primarily in the MAPK pathway and include its downstream signaling, including *NRAS*, *BRAF*, *MEK1*, *MEK2*, *NF1*, and *MITF*. Less common alterations were observed in *PIK3CA*, *PTEN*, *PIK3R1*, *HOXD8*, or *RAC1*. Interestingly, all NRAS mutations were seen in patients with acquired mutations who had been on therapy for more than 12 weeks [99].

Other pathways also remain very important as a way to bypass the inhibition of the MAPK pathway by BRAF inhibitors and confer resistance. PI3K-AKT is one such pathway and has cross-talk with the MAPK pathway. AKT can activate ERK downstream from MEK and hence avoid inhibition by both BRAF and MEK inhibitors [100] (refer to Figure 1 for various downstream signaling pathways). The work by Atefi and colleagues looked at the induction and positive feedback of AKT signaling by inhibiting the MAPK pathway and, in turn, leading to its paradoxical activation. Using in vitro methods, this resistance was at least partially reversed by using AKT inhibitors or rapamycin [101]. Luo et al. showed that the levels of phosphorylated AKT were increased in melanoma cells after treatment with vemurafenib, and higher levels were seen in vemurafenib-resistant melanoma cells [102]. Multiple receptor tyrosine kinases (RTKs) are also involved in increased activation of the MAPK pathway. This includes the platelet-derived growth factor receptor beta (PDGFRβ), insulin-like growth factor-1 (IGF-1), hepatocyte growth factor (HGF), and AXL receptors. Some of these resistance mechanisms are mediated by a nongenetic pathway. The secretion of HGF by the tumor microenvironment has been shown to cause the activation of the MAPK and PI3K-ATK pathways through the MET RTK [103]. There is also an upregulation of these various RTKs, such as AXL, EGFR, and PDGFRβ, caused by BRAF inhibitors that act in an upstream manner, causing resistance [104]. Another level at which this resistance is mediated is by transcription factors such as MITF (microphthalmia-associated transcription factor), which play a critical role in the differentiation of melanocytes and offer a unique resistance mechanism against BRAF and MEK inhibitors in malignant melanoma. There are phenotypes associated with both high and low expression of *MITF* that lead to resistant states, act as a switch between invasive and proliferative stages, and offer an example of tumor plasticity [105,106]. STAT3 (signal transducer and activator of transcription 3) is another important driver for oncogenic stimulation via upregulation of the *Mcl-1* (induced myeloid leukemia cell differentiation protein) gene, and its suppression has been shown to be effective in vemurafenib-resistant and -sensitive cells [107].

As evident from the discussion above, the resistance mechanisms to BRAF/MEK inhibitors are complicated and remain incompletely understood. This is unlike some other resistance mechanisms, such as T315I in the treatment of chronic myeloid leukemia using tyrosine kinase inhibitors, and makes overcoming the mechanism more challenging. Nevertheless, there are a variety of ongoing clinical trials aimed at overcoming these resistance mechanisms, as outlined in Table 2. These include combining BRAF/MEK inhibitors with other pro-apoptotic targets, such as inhibitors of BCL-2 (B cell lymphoma 2), CDK4/6, histone deacetylase (HDAC), and heat shock protein 90 (HSP 90). Another area of research has been the inhibition of the PIK3-AKT pathway using mTOR inhibitors and PI3K inhibitors. Lapatinib is also being evaluated as an inhibitor of RTK to prevent an increase in their signaling at the upstream level. These approaches are still mostly in the trial phase, with benefits not yet evident in clinical practice. This is owed, to some degree, to the fact that tumor cells acquire multiple resistance mechanisms, and targeting one of the pathways may not be enough. At the same time, BRAF/MEK inhibitors come with their own toxicities, and combining them with other agents will require close attention to the side effect profile.

## 7. Novel Agents and Current Ongoing Clinical Trials

Current FDA-approved BRAF/MEK inhibitors are being further explored in different treatment combinations across various cancers. At the same time, several novel BRAF and MEK inhibitors—such as VS6766, FORE8394, DS03090629, PLX8394, and BDTX4933, which can overcome paradoxical MAPK pathway activations—are currently being explored in phase I/II trials. Agents like FORE8394, PLX8394, and BDTX4933 can target both *V600* and non*V600-BRAF* mutations. Agents with good CNS penetration, such as DAY101, PF07284890, ABM-1310, and BDTX4933, are also being explored [109,110].

VS6766 (avutometinib) is a RAF/MEK clamp that inhibits BRAF, CRAF, and MEK by trapping them in inactive complexes. It is a potent inhibitor of the MAPK pathway, given that it also blocks feedback reactivation via MEK signaling [111]. It is currently being evaluated in combination with FAK inhibitor defactinib in advanced KRAS-mutated NSCLC patients after failure of prior platinum-based chemotherapy and ICI, in combination with cetuximab in KRAS-mutant mCRC, as well as in combination with defactinib in recurrent LGSOC [111,112,113,114].

DAY101 (Tovorafenib) is an oral, selective, small-molecule, type II pan-RAF inhibitor with good CNS penetration [115]. It has been evaluated in the phase II FIREFLY-1 trial in patients with R/R pediatric LGG, showing an ORR of 51% with a median DOR of 13.8 months [115]. The most common adverse effects were hair color changes, elevated serum creatinine phosphokinase, and anemia [115]. It is being further evaluated in the phase III FIREFLY-2 trial in pediatric and young adult patients with newly diagnosed LGG harboring activating *RAF* alterations [116]. Given its good CNS penetration, it has potential to treat CNS tumors and brain metastases. It was also evaluated in adult patients with R/R advanced solid tumors in dose escalation and expansion studies. It showed responses in 15% of 68 patients, with responses seen in 50% of patients with MAPK inhibitor-naive BRAF-mutated melanoma [117].

PF07284890 is another novel oral small molecule, a high CNS penetrant, and a potent BRAF inhibitor [118]. It has been shown to inhibit BRAF and CRAF in vitro studies and inhibit both *BRAFV600E* and *BRAFV600K* mutations. It demonstrated significant and durable responses in the intracranial A375-luc *BRAFV600E* melanoma xenograft model, thus showing its potential as a promising agent for CNS tumors [118]. It is currently being investigated in a phase Ia/b trial with or without binimetinib in patients with *BRAFV600*-mutant advanced solid tumors with or without brain metastasis [110].

ABM1310 is a novel small-molecule BRAF inhibitor that has been shown to have high water solubility and blood–brain barrier penetration in preclinical studies [119]. It was evaluated first in the human phase I trial of adult patients with advanced *BRAFV600*-mutated solid tumors, including those with an active brain metastasis or primary CNS cancer, regardless of prior BRAF inhibitor use [119]. The most common adverse events were skin rashes and QT prolongation. It had potential for use in patients with *BRAFV600*-mutated tumors, especially primary CNS cancers, and patients with refractory prior BRAF/MEK inhibitors [119].

BGB3245 is a RAF dimer inhibitor that has been evaluated in a phase Ia/Ib trial of 42 patients with advanced refractory tumors harboring MAPK pathway alterations with a median of three prior lines of treatment. It demonstrated 1 CR and 7 PR in 33 evaluable patients [120]. The most common adverse events were rash acneiform, maculopapular rash, fever and alanine aminotransferase (ALT) elevation, and nausea [120].

FORE8394 is a novel class I and II BRAF inhibitor that did not show feedback reactivation of the MAPK pathway in preclinical studies. It was evaluated in the phase I/IIa trial of previously treated *BRAF*-altered advanced solid and CNS tumors. It demonstrated antitumor activity in gliomas, ovarian, CRC, small bowel cancers, PTC, and ATC. PR was seen in 39% of MAPK inhibitor naive V600 mutant tumors and 18% of V600 mutated tumors with prior MAPK inhibitors. The most common adverse events were increased ALT, aspartate aminotransferase (AST), fatigue, nausea, diarrhea, and vomiting, and the most common grade 3 adverse events included increased ALT, bilirubin, and hyponatremia [121].

DS03090629 is a novel oral ATP-competitive MEK inhibitor. It has been shown to overcome acquired resistance driven by feedback reactivation of the MAPK pathway in a *BRAF*-overexpressing melanoma cell line model that was resistant to dabrafenib and trametinib in preclinical studies, making it a potential therapeutic choice for patients with resistance to BRAF and MEK inhibitors [122].

PLX8394 is another novel oral small-molecule BRAF inhibitor that blocks both monomeric BRAFV600 and dimeric nonV600 BRAF proteins without causing paradoxical MAPK pathway activation. In a phase I/II trial of PLX8394 together with cobicistat (a CYP3A4 inhibitor to enhance PLX8394 exposure), it was well tolerated and demonstrated some promising activity with 23% PR, including one ovarian cancer patient previously treated with a BRAF/MEK inhibitor [123].

BDTX4933 is a potent, highly CNS-penetrant, oral RAF inhibitor that can target class I/II/III *BRAF* mutations as well as RAF mutations. It can inhibit the MAPK pathway without paradoxical MAPK pathway activation, thus causing sustained inhibition of cellular proliferation and growth [124]. It has shown promising activity in preclinical studies and has to be further investigated in phase I studies to evaluate its use [124].

E6201 is an intravenous ATP-competitive MEK1 inhibitor that has been shown to have responses in *BRAFV600E*-mutated metastatic melanoma with brain metastasis [125]. In the phase I trial of advanced solid tumors, one patient with *BRAF*-mutated PTC during part A and three patients, including two *BRAF*-mutated melanoma and one *BRAF* wild-type melanoma in part B, had PR [126]. One patient with melanoma and brain metastasis had a near-complete response to E6201 in the initial phase I study and survived beyond 8 years [125].

There are several ongoing trials investigating MAPK pathway inhibitors, and some of those trials are listed in Table 2 (retrieved from clinicaltrials.gov) [108].

## 8. Conclusions

BRAF/MEK pathway inhibitors were initially heavily investigated and approved for the treatment of melanomas; however, they have been proven to be effective across various types of cancer with specific alternations over the past decade. Targeting the BRAF/MEK pathway is a great example of tumor agnostic therapy and precision medicine, as tumors are no longer treated or classified based on location alone but instead according to their molecular profiles. Since pembrolizumab was approved for tumors with high tumor mutation burden and MSI status, followed by the approval of larotrectinib for *NTRK* fusion, more targeted therapies are being investigated for tumor agonistic indications. Therefore, identifying the genetic aberration driving the tumor and finding the proper therapy for that aberration should now be the new direction in cancer treatment investigations. These developments have paved the way for the utilization of targeted therapies, including BRAF/MEK inhibitors, for specific genetic alterations across various cancers. At the same time, better agents to target this pathway must be investigated because most BRAF/MEK inhibitors have PFS for around one year despite their efficacy in various trials, as discussed above in this review. Very rarely, survival extends beyond two years unless these agents are combined with immunotherapy. There is also a need to further identify agents that improve the toxicity profile. Most of these combinations do not cause major or life-threatening toxicities, but side effects like pyrexia or diarrhea always affect the quality of life of the patients. In addition, further exploration of novel agents with good brain penetration as well as agents that can overcome acquired resistance by not having paradoxical MAPK pathway reactivation is needed. In summary, BRAF/MEK-targeted therapies are an important part of personalized medicine and pave the way for treatments targeting specific genetic alterations. BRAF/MEK inhibitors have shown some promising activities across various malignancies with MAPK pathway alterations; however, further investigations are needed to find better treatments that have longer duration of responses and longer survival with more tolerable toxicities that would improve the quality of life of cancer patients.

## Figures and Tables

**Table 2:** Current ongoing clinical trials.

Agent	Trial	Status	Conditions	Phase
Vemurafenib + cobimetinib	NCT05768178	R	Arm 5: adult patients with *BRAF*-positive solid and hematology malignancies (DETERMINE trial)	II/III
Vemurafenib + cobimetinib + atezolizumab	NCT02908672	A, NR	Previously untreated *BRAFV600*-mutated patients with metastatic or unresectable locally advanced melanoma	III
Dabrafenib + trametinib	NCT04940052	R	Previously treated patients with locally advanced or metastatic, radioactive iodine refractory *BRAFV600E*-mutated differentiated thyroid cancer	III
Dabrafenib + trametinib	NCT03340506	R	Rollover study, patients with solid tumors, melanoma, NSCLC, high-grade gliomas	IV
Encorafenib + binimetinib	NCT05270044	R	High-risk patients with stage II melanoma with *BRAF*-mutations as adjuvant treatment (COLUMBUS-AD)	III
Encorafenib + cetuximab +/− chemotherapy	NCT04607421	R	Previously untreated metastatic CRC (BREAKWATER)	III
Encorafenib + cetuximab + pembrolizumab vs. pembrolizumab alone	NCT05217446	R	Previously untreated *BRAFV600E* mutant, MSI high/DMMR metastatic CRC (SEAMARK)	II
Encorafenib + binimetinib + pembrolizumab	NCT04657991	R	Treatment-naive patients with advanced or metastatic melanoma with *BRAF* alterations (STEABOARD)	III
Encorafenib + binimetinib + palbociclib	NCT04720768	R	*BRAF* mutant metastatic melanoma (CELEBRATE)	I/II
Encorafenib + binimetinib +/− nivolumab	NCT04061980	R	Patients with metastatic radioiodine refractory *BRAFV600* mutant thyroid cancer	II
Trametinib vs. Standard of care	NCT02101788	A, NR	Recurrent or progressive low-grade ovarian cancer or peritoneal cavity cancer	II/III
Binimetinib + palbociclib	NCT03170206	R	Patients with advanced *KRAS* mutant NSCLC	I/II
Binimetinib + pembrolizumab	NCT03991819	R	Advanced NSCLC	I
Cobimetinib	NCT04409639	R	Newly diagnosed or HMA-treated CMML patients with RAS pathway mutations (CONCERTO trial)	II
Cobimetinib + atezolizumab	NCT04216953	R	Locally advanced and/or metastatic soft tissue sarcoma (COTESARC)	I/II
**Novel Drugs**				
BDTX-4933	NCT05786924	R	Patients with *BRAF* and other *RAS*/MAPK mutation-positive neoplasms	I
ABM-1310 +/− cobimetinib	NCT04190628	R	Patients with *BRAF*-mutated advanced solid tumors	I
BGB-3245 + mirdametinib	NCT05580770	R	Advanced metastatic or unresectable solid cancers with at least one prior line	I
Tovorafenib (DAY101) +/− pimasertib	NCT04985604	R	Recurrent, progressive, or refractory solid tumors harboring MAPK pathway aberrations	I
PF07284890	NCT05538130	R	Advanced solid tumors for which available treatments are no longer effective	I
VS6766 +/− everolimus	NCT02407509	R	Advanced solid tumors with *BRAF*, *KRAS*, and/or *NRAS* mutations	I
VS6766 + defactinib	NCT05512208	R	Recurrent gynecological cancers (endometrioid cancer, mucinous ovarian cancer, high-grade serous ovarian cancer, or cervical cancer)	II
VS6766 + sotorasib	NCT05074810	R	Previously treated *KRASG12C*-mutated NSCLC patients +/− prior G12C inhibitor (RAMP203)	I/II
VS6766 + cetuximab	NCT05200442	R	Previously treated *KRAS* mutant advanced CRC patients	I/II
E6201 + Dabrafenib	NCT05388877	R	*BRAFV600* mutated metastatic with CNS metastasis	I

R = recruiting, A, NR = active, non-recruiting. Retrieved from clinicaltrials.gov [108] assessed on 18 November 2023.

## Data Availability

Not applicable.

## References

[1] Schubert L., Mariko M.L., Clerc J., Huillard O., Groussin L. (2023). MAPK Pathway Inhibitors in Thyroid Cancer: Preclinical and Clinical Data. Cancers.

[2] Sforza V., Palumbo G., Cascetta P., Carillio G., Manzo A., Montanino A., Sandomenico C., Costanzo R., Esposito G., Laudato F. (2022). BRAF Inhibitors in Non-Small Cell Lung Cancer. Cancers.

[3] Dillon M., Lopez A., Lin E., Sales D., Perets R., Jain P. (2021). Progress on Ras/MAPK Signaling Research and Targeting in Blood and Solid Cancers. Cancers.

[4] Bouchè V., Aldegheri G., Donofrio C.A., Fioravanti A., Roberts-Thomson S., Fox S.B., Schettini F., Generali D. (2021). BRAF Signaling Inhibition in Glioblastoma: Which Clinical Perspectives?. Front. Oncol..

[5] Gouda M.A., Subbiah V. (2023). Precision oncology for BRAF-mutant cancers with BRAF and MEK inhibitors: From melanoma to tissue-agnostic therapy. ESMO Open.

[6] Pugh T.J., Bell J.L., Bruce J.P., Doherty G.J., Galvin M., Green M.F., Hunter-Zinck H., Kumari P., Lenoue-Newton M.L., Li M.M. (2022). AACR Project GENIE: 100,000 Cases and Beyond. Cancer Discov..

[7] Szklener K., Mazurek M., Wieteska M., Wacławska M., Bilski M., Mańdziuk S. (2022). New Directions in the Therapy of Glioblastoma. Cancers.

[8] Savoia P., Fava P., Casoni F., Cremona O. (2019). Targeting the ERK Signaling Pathway in Melanoma. Int. J. Mol. Sci..

[9] Chapman P.B., Hauschild A., Robert C., Haanen J.B., Ascierto P., Larkin J., Dummer R., Garbe C., Testori A., Maio M. (2011). Improved Survival with Vemurafenib in Melanoma with BRAF V600E Mutation. N. Engl. J. Med..

[10] Drugs.com FDA Approves Zelboraf and Companion Diagnostic Test for Late-Stage Skin Cancer. https://www.drugs.com/newdrugs/fda-approves-zelboraf-companion-diagnostic-test-late-stage-skin-cancer-2814.html.

[11] Drugs.com FDA Approves Tafinlar (Dabrafenib) for Advanced Melanoma. https://www.drugs.com/newdrugs/fda-approves-tafinlar-dabrafenib-advanced-melanoma-3797.html.

[12] Chapman P.B., Robert C., Larkin J., Haanen J.B., Ribas A., Hogg D., Hamid O., Ascierto P.A., Testori A., Lorigan P.C. (2017). Vemurafenib in patients with BRAFV600 mutation-positive metastatic melanoma: Final overall survival results of the randomized BRIM-3 study. Ann. Oncol..

[13] McArthur G.A., Maio M., Arance A., Nathan P., Blank C., Avril M.F., Garbe C., Hauschild A., Schadendorf D., Hamid O. (2017). Vemurafenib in metastatic melanoma patients with brain metastases: An open-label, single-arm, phase 2, multicentre study. Ann. Oncol..

[14] Hauschild A., Grob J.-J., Demidov L.V., Jouary T., Gutzmer R., Millward M., Rutkowski P., Blank C.U., Miller W.H., Kaempgen E. (2012). Dabrafenib in BRAF-mutated metastatic melanoma: A multicentre, open-label, phase 3 randomised controlled trial. Lancet.

[15] Hauschild A., Grob J.J., Demidov L.V., Jouary T., Gutzmer R., Millward M., Rutkowski P., Blank C.U., Miller W.H., Kaempgen E. (2013). An update on BREAK-3, a phase III, randomized trial: Dabrafenib (DAB) versus dacarbazine (DTIC) in patients with BRAF V600E-positive mutation metastatic melanoma (MM). J. Clin. Oncol..

[16] Long G.V., Trefzer U., Davies M.A., Kefford R.F., Ascierto P.A., Chapman P.B., Puzanov I., Hauschild A., Robert C., Algazi A. (2012). Dabrafenib in patients with Val600Glu or Val600Lys BRAF-mutant melanoma metastatic to the brain (BREAK-MB): A multicentre, open-label, phase 2 trial. Lancet Oncol..

[17] Flaherty K.T., Robert C., Hersey P., Nathan P., Garbe C., Milhem M., Demidov L.V., Hassel J.C., Rutkowski P., Mohr P. (2012). Improved Survival with MEK Inhibition in BRAF-Mutated Melanoma. N. Engl. J. Med..

[18] Drugs.com FDA Approves Mekinist (Trametinib) for Advanced Melanoma. https://www.drugs.com/newdrugs/fda-approves-mekinist-trametinib-advanced-melanoma-3798.html.

[19] Robert C., Flaherty K., Nathan P., Hersey P., Garbe C., Milhem M., Demidov L., Mohr P., Hassel J.C., Rutkowski P. (2019). Five-year outcomes from a phase 3 METRIC study in patients with BRAF V600 E/K–mutant advanced or metastatic melanoma. Eur. J. Cancer.

[20] Long G.V., Stroyakovskiy D., Gogas H., Levchenko E., De Braud F., Larkin J., Garbe C., Jouary T., Hauschild A., Grob J.J. (2014). Combined BRAF and MEK Inhibition versus BRAF Inhibition Alone in Melanoma. N. Engl. J. Med..

[21] Drugs.com GSK Gains Accelerated FDA Approval for Combination Use of Mekinist (Trametinib) and Tafinlar (Dabrafenib). https://www.drugs.com/newdrugs/gsk-gains-accelerated-fda-approval-combination-mekinist-trametinib-tafinlar-dabrafenib-4003.html.

[22] Drugs.com FDA Approves Cotellic (Cobimetinib) for the Combination Treatment of Advanced Melanoma. https://www.drugs.com/newdrugs/fda-approves-cotellic-cobimetinib-combination-advanced-melanoma-4295.html.

[23] Long G.V., Flaherty K.T., Stroyakovskiy D., Gogas H., Levchenko E., de Braud F., Larkin J., Garbe C., Jouary T., Hauschild A. (2017). Dabrafenib plus trametinib versus dabrafenib monotherapy in patients with metastatic BRAF V600E/K-mutant melanoma: Long-term survival and safety analysis of a phase 3 study. Ann. Oncol..

[24] Robert C., Karaszewska B., Schachter J., Rutkowski P., Mackiewicz A., Stroiakovski D., Lichinitser M., Dummer R., Grange F., Mortier L. (2015). Improved Overall Survival in Melanoma with Combined Dabrafenib and Trametinib. N. Engl. J. Med..

[25] Davies M.A., Saiag P., Robert C., Grob J.-J., Flaherty K.T., Arance A., Chiarion-Sileni V., Thomas L., Lesimple T., Mortier L. (2017). Dabrafenib plus trametinib in patients with BRAFV600-mutant melanoma brain metastases (COMBI-MB): A multicentre, multicohort, open-label, phase 2 trial. Lancet Oncol..

[26] Long G.V., Hauschild A., Santinami M., Atkinson V., Mandalà M., Chiarion-Sileni V., Larkin J., Nyakas M., Dutriaux C., Haydon A. (2017). Adjuvant Dabrafenib plus Trametinib in Stage III BRAF-Mutated Melanoma. N. Engl. J. Med..

[27] Drugs.com Novartis Receives FDA Approval of Tafinlar + Mekinist for Adjuvant Treatment of BRAF V600-Mutant Melanoma. https://www.drugs.com/newdrugs/novartis-receives-fda-approval-tafinlar-mekinist-adjuvant-braf-v600-mutant-melanoma-4733.html.

[28] Larkin J., Ascierto P.A., Dréno B., Atkinson V., Liszkay G., Maio M., Mandalà M., Demidov L., Stroyakovskiy D., Thomas L. (2014). Combined Vemurafenib and Cobimetinib in BRAF-Mutated Melanoma. N. Engl. J. Med..

[29] Ascierto P.A., McArthur G.A., Dréno B., Atkinson V., Liszkay G., Di Giacomo A.M., Mandalà M., Demidov L., Stroyakovskiy D., Thomas L. (2016). Cobimetinib combined with vemurafenib in advanced BRAFV600-mutant melanoma (coBRIM): Updated efficacy results from a randomised, double-blind, phase 3 trial. Lancet Oncol..

[30] Ascierto P.A., Dréno B., Larkin J., Ribas A., Liszkay G., Maio M., Mandalà M., Demidov L., Stroyakovskiy D., Thomas L. (2021). 5-Year Outcomes with Cobimetinib plus Vemurafenib in BRAFV600 Mutation–Positive Advanced Melanoma: Extended Follow-up of the coBRIM Study. Clin. Cancer Res..

[31] Dummer R., Ascierto P.A., Gogas H.J., Arance A., Mandala M., Liszkay G., Garbe C., Schadendorf D., Krajsova I., Gutzmer R. (2018). Encorafenib plus binimetinib versus vemurafenib or encorafenib in patients with BRAF-mutant melanoma (COLUMBUS): A multicentre, open-label, randomised phase 3 trial. Lancet Oncol..

[32] Drugs.com Array BioPharma Announces FDA Approval of Braftovi (Encorafenib) in Combination with Mektovi (Binimetinib) for Unresectable or Metastatic Melanoma with BRAF Mutations. https://www.drugs.com/newdrugs/array-biopharma-announces-fda-approval-braftovi-encorafenib-combination-mektovi-binimetinib-4771.html.

[33] Dummer R., Ascierto P.A., Gogas H.J., Arance A., Mandala M., Liszkay G., Garbe C., Schadendorf D., Krajsova I., Gutzmer R. (2018). Overall survival in patients with BRAF-mutant melanoma receiving encorafenib plus binimetinib versus vemurafenib or encorafenib (COLUMBUS): A multicentre, open-label, randomised, phase 3 trial. Lancet Oncol..

[34] Dummer R., Flaherty K.T., Robert C., Arance A., De Groot J.W.B., Garbe C., Gogas H.J., Gutzmer R., Krajsová I., Liszkay G. (2022). COLUMBUS 5-Year Update: A Randomized, Open-Label, Phase III Trial of Encorafenib Plus Binimetinib Versus Vemurafenib or Encorafenib in Patients with *BRAF* V600–Mutant Melanoma. J. Clin. Oncol..

[35] Salama A.K.S., Li S., Macrae E.R., Park J.-I., Mitchell E.P., Zwiebel J.A., Chen H.X., Gray R.J., McShane L.M., Rubinstein L.V. (2020). Dabrafenib and Trametinib in Patients with Tumors with BRAFV600E Mutations: Results of the NCI-MATCH Trial Subprotocol H. J. Clin. Oncol..

[36] Kopetz S., Grothey A., Yaeger R., Van Cutsem E., Desai J., Yoshino T., Wasan H., Ciardiello F., Loupakis F., Hong Y.S. (2019). Encorafenib, Binimetinib, and Cetuximab in BRAF V600E–Mutated Colorectal Cancer. N. Engl. J. Med..

[37] Tabernero J., Grothey A., Van Cutsem E., Yaeger R., Wasan H., Yoshino T., Desai J., Ciardiello F., Loupakis F., Hong Y.S. (2021). Encorafenib Plus Cetuximab as a New Standard of Care for Previously Treated BRAF V600E–Mutant Metastatic Colorectal Cancer: Updated Survival Results and Subgroup Analyses from the BEACON Study. J. Clin. Oncol..

[38] Van Cutsem E., Taieb J., Yaeger R., Yoshino T., Grothey A., Maiello E., Elez E., Dekervel J., Ross P., Ruiz-Casado A. (2023). ANCHOR CRC: Results From a Single-Arm, Phase II Study of Encorafenib Plus Binimetinib and Cetuximab in Previously Untreated BRAF(V600E)-Mutant Metastatic Colorectal Cancer. J. Clin. Oncol..

[39] Subbiah V., Puzanov I., Blay J.-Y., Chau I., Lockhart A.C., Raje N.S., Wolf J., Baselga J., Meric-Bernstam F., Roszik J. (2020). Pan-Cancer Efficacy of Vemurafenib in BRAFV600-Mutant Non-Melanoma Cancers. Cancer Discov..

[40] Kaley T., Touat M., Subbiah V., Hollebecque A., Rodon J., Lockhart A.C., Keedy V., Bielle F., Hofheinz R.-D., Joly F. (2018). BRAF Inhibition in BRAFV600-Mutant Gliomas: Results From the VE-BASKET Study. J. Clin. Oncol..

[41] Subbiah V., Gervais R., Riely G., Hollebecque A., Blay J.-Y., Felip E., Schuler M., Gonçalves A., Italiano A., Keedy V. (2019). Efficacy of Vemurafenib in Patients with Non–Small-Cell Lung Cancer with BRAFV600 Mutation: An Open-Label, Single-Arm Cohort of the Histology-Independent VE-BASKET Study. JCO Precis. Oncol..

[42] Diamond E.L., Subbiah V., Lockhart A.C., Blay J.-Y., Puzanov I., Chau I., Raje N.S., Wolf J., Erinjeri J.P., Torrisi J. (2018). Vemurafenib for *BRAF* V600–Mutant Erdheim-Chester Disease and Langerhans Cell Histiocytosis. JAMA Oncol..

[43] Hyman D.M., Puzanov I., Subbiah V., Faris J.E., Chau I., Blay J.-Y., Wolf J., Raje N.S., Diamond E.L., Hollebecque A. (2015). Vemurafenib in Multiple Nonmelanoma Cancers with *BRAF* V600 Mutations. N. Engl. J. Med..

[44] Subbiah V., Kreitman R.J., Wainberg Z.A., Gazzah A., Lassen U., Stein A., Wen P.Y., Dietrich S., De Jonge M.J.A., Blay J.-Y. (2023). Dabrafenib plus trametinib in BRAFV600E-mutated rare cancers: The phase 2 ROAR trial. Nat. Med..

[45] Kreitman R.J., Moreau P., Ravandi F., Hutchings M., Gazzah A., Michallet A.-S., Wainberg Z.A., Stein A., Dietrich S., de Jonge M.J.A. (2023). Dabrafenib plus trametinib in patients with relapsed/refractory BRAF V600E mutation–positive hairy cell leukemia. Blood.

[46] Planchard D., Besse B., Groen H.J.M., Hashemi S.M.S., Mazieres J., Kim T.M., Quoix E., Souquet P.-J., Barlesi F., Baik C. (2022). Phase 2 Study of Dabrafenib Plus Trametinib in Patients with BRAF V600E-Mutant Metastatic NSCLC: Updated 5-Year Survival Rates and Genomic Analysis. J. Thorac. Oncol..

[47] Planchard D., Besse B., Groen H.J.M., Souquet P.-J., Quoix E., Baik C.S., Barlesi F., Kim T.M., Mazieres J., Novello S. (2016). Dabrafenib plus trametinib in patients with previously treated BRAFV600E-mutant metastatic non-small cell lung cancer: An open-label, multicentre phase 2 trial. Lancet Oncol..

[48] Planchard D., Smit E.F., Groen H.J.M., Mazieres J., Besse B., Helland Å., Giannone V., D’Amelio A.M., Zhang P., Mookerjee B. (2017). Dabrafenib plus trametinib in patients with previously untreated BRAFV600E-mutant metastatic non-small-cell lung cancer: An open-label, phase 2 trial. Lancet Oncol..

[49] Mazieres J., Cropet C., Montané L., Barlesi F., Souquet P.J., Quantin X., Dubos-Arvis C., Otto J., Favier L., Avrillon V. (2020). Vemurafenib in non-small-cell lung cancer patients with BRAFV600 and BRAFnonV600 mutations. Ann. Oncol..

[50] Riely G.J., Smit E.F., Ahn M.-J., Felip E., Ramalingam S.S., Tsao A., Johnson M., Gelsomino F., Esper R., Nadal E. (2023). Phase II, Open-Label Study of Encorafenib Plus Binimetinib in Patients with BRAFV600-Mutant Metastatic Non–Small-Cell Lung Cancer. J. Clin. Oncol..

[51] Diamond E.L., Durham B.H., Ulaner G.A., Drill E., Buthorn J., Ki M., Bitner L., Cho H., Young R.J., Francis J.H. (2019). Efficacy of MEK inhibition in patients with histiocytic neoplasms. Nature.

[52] Whitlock J.A., Geoerger B., Dunkel I.J., Roughton M., Choi J., Osterloh L., Russo M., Hargrave D. (2023). Dabrafenib, alone or in combination with trametinib, in BRAF V600–mutated pediatric Langerhans cell histiocytosis. Blood Adv..

[53] Gershenson D.M., Miller A., Brady W.E., Paul J., Carty K., Rodgers W., Millan D., Coleman R.L., Moore K.N., Banerjee S. (2022). Trametinib versus standard of care in patients with recurrent low-grade serous ovarian cancer (GOG 281/LOGS): An international, randomised, open-label, multicentre, phase 2/3 trial. Lancet.

[54] Ribas A., Ferrucci P.F., Atkinson V., Stephens R., Long G.V., Lawrence D.P., Del Vecchio M., Hamid O., Schmidt H., Schachter J. (2022). Pembrolizumab (pembro) plus dabrafenib (dab) and trametinib (tram) in BRAFV600E/K-mutant melanoma: Long-term follow-up of KEYNOTE-022 parts 1, 2, and 3. J. Clin. Oncol..

[55] Gutzmer R., Stroyakovskiy D., Gogas H., Robert C., Lewis K., Protsenko S., Pereira R.P., Eigentler T., Rutkowski P., Demidov L. (2020). Atezolizumab, vemurafenib, and cobimetinib as first-line treatment for unresectable advanced BRAF(V600) mutation-positive melanoma (IMspire150): Primary analysis of the randomised, double-blind, placebo-controlled, phase 3 trial. Lancet.

[56] Ros J., Baraibar I., Sardo E., Mulet N., Salvà F., Argilés G., Martini G., Ciardiello D., Cuadra J.L., Tabernero J. (2021). BRAF, MEK and EGFR inhibition as treatment strategies in BRAF V600E metastatic colorectal cancer. Ther. Adv. Med. Oncol..

[57] Drugs.com FDA Approves Braftovi (Encorafenib) in Combination with Cetuximab for the Treatment of BRAFV600E-Mutant Metastatic Colorectal Cancer (CRC) after Prior Therapy. https://www.drugs.com/newdrugs/fda-approves-braftovi-encorafenib-combination-cetuximab-brafv600e-mutant-metastatic-colorectal-5201.html.

[58] Van Cutsem E., Huijberts S., Grothey A., Yaeger R., Cuyle P.-J., Elez E., Fakih M., Montagut C., Peeters M., Yoshino T. (2019). Binimetinib, Encorafenib, and Cetuximab Triplet Therapy for Patients with BRAF V600E–Mutant Metastatic Colorectal Cancer: Safety Lead-In Results From the Phase III BEACON Colorectal Cancer Study. J. Clin. Oncol..

[59] Kopetz S., Grothey A., Yaeger R., Ciardiello F., Desai J., Kim T.W., Maughan T., Cutsem E.V., Wasan H.S., Yoshino T. (2021). BREAKWATER: Randomized phase 3 study of encorafenib (enco) + cetuximab (cetux) ± chemotherapy for first-line (1L) treatment (tx) of BRAF V600E-mutant (BRAFV600E) metastatic colorectal cancer (mCRC). J. Clin. Oncol..

[60] Lamarca A., Edeline J., Goyal L. (2022). How I treat biliary tract cancer. ESMO Open.

[61] Subbiah V., Lassen U., Élez E., Italiano A., Curigliano G., Javle M., de Braud F., Prager G.W., Greil R., Stein A. (2020). Dabrafenib plus trametinib in patients with BRAFV600E-mutated biliary tract cancer (ROAR): A phase 2, open-label, single-arm, multicentre basket trial. Lancet Oncol..

[62] Jain A., Kwong L.N., Javle M. (2016). Genomic Profiling of Biliary Tract Cancers and Implications for Clinical Practice. Curr. Treat. Options Oncol..

[63] Drugs.com Novartis Tafinlar + Mekinist Receives FDA Approval for First Tumor-Agnostic Indication for BRAF V600E Solid Tumors. https://www.drugs.com/newdrugs/novartis-tafinlar-mekinist-receives-fda-approval-first-tumor-agnostic-indication-braf-v600e-solid-5857.html.

[64] Negrao M.V., Raymond V.M., Lanman R.B., Robichaux J.P., He J., Nilsson M.B., Ng P.K.S., Amador B.E., Roarty E.B., Nagy R.J. (2020). Molecular Landscape of BRAF-Mutant NSCLC Reveals an Association Between Clonality and Driver Mutations and Identifies Targetable Non-V600 Driver Mutations. J. Thorac. Oncol..

[65] Guaitoli G., Zullo L., Tiseo M., Dankner M., Rose A.A., Facchinetti F. (2023). Non-small-cell lung cancer: How to manage BRAF-mutated disease. Drugs Context.

[66] Dudnik E., Peled N., Nechushtan H., Wollner M., Onn A., Agbarya A., Moskovitz M., Keren S., Popovits-Hadari N., Urban D. (2018). BRAF Mutant Lung Cancer: Programmed Death Ligand 1 Expression, Tumor Mutational Burden, Microsatellite Instability Status, and Response to Immune Check-Point Inhibitors. J. Thorac. Oncol..

[67] Reddy V.P., Gay L.M., Elvin J.A., Vergilio J.-A., Suh J., Ramkissoon S., Daniel S., Severson E.A., Ali S.M., Schrock A.B. (2017). BRAF fusions in clinically advanced non-small cell lung cancer: An emerging target for anti-BRAF therapies. J. Clin. Oncol..

[68] Arcila M.E., Drilon A., Sylvester B.E., Lovly C.M., Borsu L., Reva B., Kris M.G., Solit D.B., Ladanyi M. (2015). MAP2K1 (MEK1) Mutations Define a Distinct Subset of Lung Adenocarcinoma Associated with Smoking. Clin. Cancer Res..

[69] FDA.gov FDA Grants Regular Approval to Dabrafenib and Trametinib Combination for Metastatic NSCLC with BRAF V600E Mutation. https://www.fda.gov/drugs/resources-information-approved-drugs/fda-grants-regular-approval-dabrafenib-and-trametinib-combination-metastatic-nsclc-braf-v600e.

[70] Garutti M., Bergnach M., Polesel J., Palmero L., Pizzichetta M.A., Puglisi F. (2022). BRAF and MEK Inhibitors and Their Toxicities: A Meta-Analysis. Cancers.

[71] Drugs.com FDA Approves Braftovi (Encorafenib) with Mektovi (Binimetinib) for Metastatic Non-Small Cell Lung Cancer with a BRAF V600E Mutation. https://www.drugs.com/newdrugs/fda-approves-braftovi-encorafenib-mektovi-binimetinib-metastatic-non-small-cell-lung-cancer-braf-6111.html.

[72] Tiacci E., Trifonov V., Schiavoni G., Holmes A., Kern W., Martelli M.P., Pucciarini A., Bigerna B., Pacini R., Wells V.A. (2011). BRAF Mutations in Hairy-Cell Leukemia. N. Engl. J. Med..

[73] Tiacci E., Schiavoni G., Forconi F., Santi A., Trentin L., Ambrosetti A., Cecchini D., Sozzi E., Francia di Celle P., Di Bello C. (2012). Simple genetic diagnosis of hairy cell leukemia by sensitive detection of the BRAF-V600E mutation. Blood.

[74] Tiacci E., Schiavoni G., Martelli M.P., Boveri E., Pacini R., Tabarrini A., Zibellini S., Santi A., Pettirossi V., Fortini E. (2013). Constant activation of the RAF-MEK-ERK pathway as a diagnostic and therapeutic target in hairy cell leukemia. Haematologica.

[75] Mason E.F., Brown R.D., Szeto D.P., Gibson C.J., Jia Y., Garcia E.P., Jacobson C.A., Dal Cin P., Kuo F.C., Pinkus G.S. (2017). Detection of activating MAP2K1 mutations in atypical hairy cell leukemia and hairy cell leukemia variant. Leuk. Lymphoma.

[76] Tiacci E., Park J.H., De Carolis L., Chung S.S., Broccoli A., Scott S., Zaja F., Devlin S., Pulsoni A., Chung Y.R. (2015). Targeting Mutant BRAF in Relapsed or Refractory Hairy-Cell Leukemia. N. Engl. J. Med..

[77] Tiacci E., De Carolis L., Simonetti E., Capponi M., Ambrosetti A., Lucia E., Antolino A., Pulsoni A., Ferrari S., Zinzani P.L. (2021). Vemurafenib plus Rituximab in Refractory or Relapsed Hairy-Cell Leukemia. N. Engl. J. Med..

[78] Aaroe A., Kurzrock R., Goyal G., Goodman A.M., Patel H., Ruan G., Ulaner G., Young J., Li Z., Dustin D. (2023). Successful treatment of non-Langerhans cell histiocytosis with the MEK inhibitor trametinib: A multicenter analysis. Blood Adv..

[79] Drugs.com FDA Approves Zelboraf (Vemurafenib) for Erdheim-Chester Disease with BRAF V600 Mutation. https://www.drugs.com/newdrugs/fda-approves-zelboraf-vemurafenib-erdheim-chester-braf-v600-mutation-4625.html.

[80] Drugs.com FDA Approves Oral MEK Inhibitor Cobimetinib for Histiocytic Neoplasms, Research Led by Memorial Sloan Kettering Cancer Center. https://www.drugs.com/newdrugs/fda-approves-oral-mek-inhibitor-cobimetinib-histiocytic-neoplasms-research-led-memorial-sloan-5984.html.

[81] Di Nunno V., Gatto L., Tosoni A., Bartolini S., Franceschi E. (2022). Implications of BRAF V600E mutation in gliomas: Molecular considerations, prognostic value and treatment evolution. Front. Oncol..

[82] Wen P.Y., Stein A., van den Bent M., De Greve J., Wick A., de Vos F.Y.F.L., von Bubnoff N., van Linde M.E., Lai A., Prager G.W. (2022). Dabrafenib plus trametinib in patients with BRAFV600E-mutant low-grade and high-grade glioma (ROAR): A multicentre, open-label, single-arm, phase 2, basket trial. Lancet Oncol..

[83] Brose M.S., Cabanillas M.E., Cohen E.E.W., Wirth L.J., Riehl T., Yue H., Sherman S.I., Sherman E.J. (2016). Vemurafenib in patients with BRAFV600E-positive metastatic or unresectable papillary thyroid cancer refractory to radioactive iodine: A non-randomised, multicentre, open-label, phase 2 trial. Lancet Oncol..

[84] Sukrithan V., Jain P., Shah M.H., Konda B. (2023). Kinase inhibitors in thyroid cancers. Endocr. Oncol..

[85] Leboulleux S., Benisvy D., Taieb D., Attard M., Bournaud C., Terroir M., Al Ghuzlan A., Lamartina L., Schlumberger M.J., Godbert Y. (2021). 1743MO MERAIODE: A redifferentiation phase II trial with trametinib followed by radioactive iodine for metastatic radioactive iodine refractory differentiated thyroid cancer patients with a RAS mutation. Ann. Oncol..

[86] Leboulleux S., Benisvy D., Taieb D., Attard M., Bournaud C., Terroir-Cassou-Mounat M., Lacroix L., Anizan N., Schiazza A., Garcia M.E. (2023). MERAIODE: A Phase II Redifferentiation Trial with Trametinib and 131 I in Metastatic Radioactive Iodine Refractory RAS Mutated Differentiated Thyroid Cancer. Thyroid.

[87] Drugs.com FDA Approves Tafinlar + Mekinist for the Treatment of BRAF-Positive Anaplastic Thyroid Cancer. https://www.drugs.com/newdrugs/fda-approves-tafinlar-mekinist-braf-positive-anaplastic-thyroid-cancer-4737.html.

[88] Subbiah V., Kreitman R.J., Wainberg Z.A., Cho J.Y., Schellens J.H.M., Soria J.C., Wen P.Y., Zielinski C., Cabanillas M.E., Urbanowitz G. (2018). Dabrafenib and Trametinib Treatment in Patients with Locally Advanced or Metastatic *BRAF* V600–Mutant Anaplastic Thyroid Cancer. J. Clin. Oncol..

[89] Grisham R.N., Vergote I., Banerjee S., Drill E., Kalbacher E., Mirza M.R., Romero I., Vuylsteke P., Coleman R.L., Hilpert F. (2023). Molecular Results and Potential Biomarkers Identified from the Phase 3 MILO/ENGOT-ov11 Study of Binimetinib versus Physician Choice of Chemotherapy in Recurrent Low-Grade Serous Ovarian Cancer. Clin. Cancer Res..

[90] Moujaber T., Balleine R.L., Gao B., Madsen I., Harnett P.R., DeFazio A. (2021). New therapeutic opportunities for women with low-grade serous ovarian cancer. Endocr. Relat. Cancer.

[91] Monk B.J., Grisham R.N., Banerjee S., Kalbacher E., Mirza M.R., Romero I., Vuylsteke P., Coleman R.L., Hilpert F., Oza A.M. (2020). MILO/ENGOT-ov11: Binimetinib Versus Physician’s Choice Chemotherapy in Recurrent or Persistent Low-Grade Serous Carcinomas of the Ovary, Fallopian Tube, or Primary Peritoneum. J. Clin. Oncol..

[92] Dummer R., Queirolo P., Gerard Duhard P., Hu Y., Wang D., de Azevedo S.J., Robert C., Ascierto P.A., Chiarion-Sileni V., Pronzato P. (2023). Atezolizumab, vemurafenib, and cobimetinib in patients with melanoma with CNS metastases (TRICOTEL): A multicentre, open-label, single-arm, phase 2 study. Lancet Oncol..

[93] Drugs.com FDA Approves Genentech’s Tecentriq plus Cotellic and Zelboraf for People with Advanced Melanoma. https://www.drugs.com/newdrugs/fda-approves-genentech-s-tecentriq-plus-cotellic-zelboraf-advanced-melanoma-5311.html.

[94] Dummer R., Long G.V., Robert C., Tawbi H.A., Flaherty K.T., Ascierto P.A., Nathan P.D., Rutkowski P., Leonov O., Dutriaux C. (2022). Randomized Phase III Trial Evaluating Spartalizumab Plus Dabrafenib and Trametinib for BRAF V600–Mutant Unresectable or Metastatic Melanoma. J. Clin. Oncol..

[95] Ferrucci P.F., Di Giacomo A.M., Del Vecchio M., Atkinson V., Schmidt H., Schachter J., Queirolo P., Long G.V., Stephens R., Svane I.M. (2020). KEYNOTE-022 part 3: A randomized, double-blind, phase 2 study of pembrolizumab, dabrafenib, and trametinib in BRAF-mutant melanoma. J. ImmunoTherapy Cancer.

[96] Schadendorf D., Dummer R., Robert C., Ribas A., Sullivan R.J., Panella T., McKean M., Santos E.S., Brill K., Polli A. (2022). STARBOARD: Encorafenib + binimetinib + pembrolizumab for first-line metastatic/unresectable BRAF V600-mutant melanoma. Future Oncol..

[97] Tangella L.P., Clark M.E., Gray E.S. (2021). Resistance mechanisms to targeted therapy in BRAF-mutant melanoma—A mini review. Biochim. Biophys. Acta (BBA)—Gen. Subj..

[98] Johnson D.B., Menzies A.M., Zimmer L., Eroglu Z., Ye F., Zhao S., Rizos H., Sucker A., Scolyer R.A., Gutzmer R. (2015). Acquired BRAF inhibitor resistance: A multicenter meta-analysis of the spectrum and frequencies, clinical behaviour, and phenotypic associations of resistance mechanisms. Eur. J. Cancer.

[99] Van Allen E.M., Wagle N., Sucker A., Treacy D.J., Johannessen C.M., Goetz E.M., Place C.S., Taylor-Weiner A., Whittaker S., Kryukov G.V. (2014). The genetic landscape of clinical resistance to RAF inhibition in metastatic melanoma. Cancer Discov..

[100] Jiang C.C., Lai F., Thorne R.F., Yang F., Liu H., Hersey P., Zhang X.D. (2011). MEK-Independent Survival of B-RAFV600E Melanoma Cells Selected for Resistance to Apoptosis Induced by the RAF Inhibitor PLX4720. Clin. Cancer Res..

[101] Atefi M., von Euw E., Attar N., Ng C., Chu C., Guo D., Nazarian R., Chmielowski B., Glaspy J.A., Comin-Anduix B. (2011). Reversing melanoma cross-resistance to BRAF and MEK inhibitors by co-targeting the AKT/mTOR pathway. PLoS ONE.

[102] Luo H., Umebayashi M., Doi K., Morisaki T., Shirasawa S., Tsunoda T. (2016). Resveratrol Overcomes Cellular Resistance to Vemurafenib Through Dephosphorylation of AKT in BRAF-mutated Melanoma Cells. Anticancer Res..

[103] Straussman R., Morikawa T., Shee K., Barzily-Rokni M., Qian Z.R., Du J., Davis A., Mongare M.M., Gould J., Frederick D.T. (2012). Tumour micro-environment elicits innate resistance to RAF inhibitors through HGF secretion. Nature.

[104] Shaffer S.M., Dunagin M.C., Torborg S.R., Torre E.A., Emert B., Krepler C., Beqiri M., Sproesser K., Brafford P.A., Xiao M. (2017). Rare cell variability and drug-induced reprogramming as a mode of cancer drug resistance. Nature.

[105] Kemper K., de Goeje P.L., Peeper D.S., van Amerongen R. (2014). Phenotype Switching: Tumor Cell Plasticity as a Resistance Mechanism and Target for Therapy. Cancer Res..

[106] Müller J., Krijgsman O., Tsoi J., Robert L., Hugo W., Song C., Kong X., Possik P.A., Cornelissen-Steijger P.D.M., Foppen M.H.G. (2014). Low MITF/AXL ratio predicts early resistance to multiple targeted drugs in melanoma. Nat. Commun..

[107] Golub K., Bai W., Zhang Z., Xiao H., Sun R., Shen J., Sun J. (2023). The mechanism and consequences of BRAF inhibitor resistance in melanoma. Genome Instab. Dis..

[108] https://clinicaltrials.gov/.

[109] Landi D.B., Ziegler D.S., Franson A.F., Baxter P.A., Leary S., Larouche V., Waanders A.J., Lugt J.V.d., McCowage G.B., Doz F. (2022). FIREFLY-1 (PNOC 026): A phase 2 study to evaluate the safety and efficacy of tovorafenib (DAY101) in pediatric patients with RAF-altered recurrent or progressive low-grade glioma or advanced solid tumors. J. Clin. Oncol..

[110] Subbiah V., Gutierrez M., Anders C.K., Ansstas G., Owonikoko T.K., Monga V., Forsyth P.A.J., Dagogo-Jack I., Chandra S., Tsai K.K. (2021). Trial in progress: Phase 1a/b study of PF-07284890 (brain-penetrant BRAF inhibitor) with/without binimetinib in patients with BRAF V600-mutant solid tumors. J. Clin. Oncol..

[111] Govindan R., Awad M.M., Gadgeel S.M., Pachter J.A., Patrick G., Denis L.J. (2022). A phase 1/2 study of VS-6766 (RAF/MEK clamp) in combination with sotorasib (G12C inhibitor) in patients with KRAS G12C mutant non–small cell lung cancer (NSCLC) (RAMP 203). J. Clin. Oncol..

[112] Banerjee S.N., Monk B.J., Nieuwenhuysen E.V., Moore K.N., Oaknin A., Fabbro M., Colombo N., O’Malley D.M., Coleman R.L., Oza A.M. (2022). ENGOT-ov60/GOG-3052/RAMP 201: A phase 2 study of VS-6766 (RAF/MEK clamp) alone and in combination with defactinib (FAK inhibitor) in recurrent low-grade serous ovarian cancer (LGSOC). J. Clin. Oncol..

[113] Capelletto E., Bironzo P., Denis L., Koustenis A., Bungaro M., Novello S. (2022). Single agent VS-6766 or VS-6766 plus defactinib in KRAS-mutant non-small-cell lung cancer: The RAMP-202 phase II trial. Future Oncol..

[114] Shergill A., Liao C.-Y., Kindler H.L., Polite B.N., Catenacci D.V.T. (2022). A phase 1b/2 study of VS-6766 in combination cetuximab in patients (pts) with advanced KRAS mt colorectal cancer (CRC). J. Clin. Oncol..

[115] Kilburn L.B., Khuong-Quang D.-A., Hansford J.R., Landi D., van der Lugt J., Leary S.E.S., Driever P.H., Bailey S., Perreault S., McCowage G. (2023). The type II RAF inhibitor tovorafenib in relapsed/refractory pediatric low-grade glioma: The phase 2 FIREFLY-1 trial. Nat. Med..

[116] Tilburg C.M.v., Kilburn L.B., Crotty E., Smith A.A., Perreault S., Franson A.F., Jabado N., Hoffman L.M., Schmidt R., Meeteren A.Y.N.S.-v. (2023). LOGGIC/FIREFLY-2: A phase 3, randomized trial of tovorafenib vs. chemotherapy in pediatric and young adult patients with newly diagnosed low-grade glioma harboring an activating RAF alteration. J. Clin. Oncol..

[117] Rasco D.W., Medina T., Corrie P., Pavlick A.C., Middleton M.R., Lorigan P., Hebert C., Plummer R., Larkin J., Agarwala S.S. (2023). Phase 1 study of the pan-RAF inhibitor tovorafenib in patients with advanced solid tumors followed by dose expansion in patients with metastatic melanoma. J. Clin. Oncol..

[118] Bouhana K., Anderson D., DeWolf W., Brown S., Williams L., Ren L., Moreno D., Wallace R., Fell J.B., Hartley D. (2021). Abstract 1473: Nonclinical development of PF-07284890 (ARRY-461), a potent, brain-penetrant, small molecule inhibitor of BRAF V600-mutation-driven tumors in vitro and in vivo. Cancer Res..

[119] Piha-Paul S.A., Nagpal S., Weise A.M., Braiteh F.S., Chen C., Huang C.Q., Liu W., Hu Y., Yang Z., Tsai K.K. (2023). A phase 1, multicenter, open-label study of a new BRAF inhibitor ABM-1310 in adult patients (pts) with BRAFv600-mutated solid tumors. J. Clin. Oncol..

[120] Schram A.M., Subbiah V., Sullivan R., Cosman R., Liu J., Sbar E.I., Hoang T., Chen J., Johnson M., Amoruccio V. (2023). Abstract CT031: A first-in-human, phase 1a/1b, open-label, dose-escalation and expansion study to investigate the safety, pharmacokinetics, and antitumor activity of the RAF dimer inhibitor BGB-3245 in patients with advanced or refractory tumors. Cancer Res..

[121] Fuente M.I.D.L., Ahnert J.R., Yaeger R., Tsai F.Y.-C., Janku F., Butowski N.A., Allen C.E., Ammakkanavar N.R., Taylor J.W., Michelson G. (2023). Safety and efficacy of the novel BRAF inhibitor FORE8394 in patients with advanced solid and CNS tumors: Results from a phase 1/2a study. J. Clin. Oncol..

[122] Takano K., Munehira Y., Hatanaka M., Murakami R., Shibata Y., Shida T., Takeuchi K., Takechi S., Tabata T., Shimada T. (2023). Discovery of a Novel ATP-Competitive MEK Inhibitor DS03090629 that Overcomes Resistance Conferred by BRAF Overexpression in BRAF-Mutated Melanoma. Mol. Cancer Ther..

[123] Janku F., Vaishampayan U.N., Khemka V., Bhatty M., Sherman E.J., Tao J., Whisenant J.R., Hong D.S., Bui N., Kummar S. (2018). Phase 1/2 precision medicine study of the next-generation BRAF inhibitor PLX8394. J. Clin. Oncol..

[124] Han Y.-C., Ng P.-Y., Ogawa L.S., Yang S.N., Chen M., Ishiyama N., Lin T.-A., Buck E. (2023). Abstract 3415: Preclinical characterization of a brain penetrant RAF inhibitor, BDTX-4933, targeting oncogenic BRAF Class I/II/III and RAS mutations. Cancer Res..

[125] Babiker H.M., Byron S.A., Hendricks W.P.D., Elmquist W.F., Gampa G., Vondrak J., Aldrich J., Cuyugan L., Adkins J., De Luca V. (2019). E6201, an intravenous MEK1 inhibitor, achieves an exceptional response in BRAF V600E-mutated metastatic malignant melanoma with brain metastases. Investig. New Drugs.

[126] Tibes R., Borad M.J., Dutcus C.E., Reyderman L., Feit K., Eisen A., Verbel D.A., Von Hoff D.D. (2018). Safety, pharmacokinetics, and preliminary efficacy of E6201 in patients with advanced solid tumours, including melanoma: Results of a phase 1 study. Br. J. Cancer.

